# The effect of changing foot progression angle using real-time visual feedback on rearfoot eversion during running

**DOI:** 10.1371/journal.pone.0246425

**Published:** 2021-02-10

**Authors:** Seyed Hamed Mousavi, Laurens van Kouwenhove, Reza Rajabi, Johannes Zwerver, Juha M. Hijmans

**Affiliations:** 1 Department of Rehabilitation Medicine, University Medical Center Groningen, University of Groningen, Groningen, The Netherlands; 2 Department of Health and Sport Medicine, Faculty of Physical Education and Sport Sciences, University of Tehran, Tehran, Iran; 3 Center for Human Movement Science, University Medical Center Groningen, University of Groningen, Groningen, The Netherlands; 4 Department of Sports Medicine, Gelderse Vallei Hospital, Ede, The Netherlands; University of Pittsburgh, UNITED STATES

## Abstract

Atypical rearfoot in/eversion may be an important risk factor for running-related injuries. Prominent interventions for atypical rearfoot eversion include foot orthoses, footwear, and taping but a modification derived from gait retraining to correct atypical rearfoot in/eversion is lacking. We aimed to investigate changes in rearfoot in/eversion, subtalar pronation, medial longitudinal arch angle, and selected lower limb joint biomechanics while performing toe-in/toe-out running using real-time visual feedback. Fifteen female runners participated in this study. Subjects performed toe-in/toe-out running using real-time visual feedback on foot progression angle, which was set ±5° from habitual foot progression angle. 3D kinematics of rearfoot in/eversion, subtalar supination/pronation, medial longitudinal arch angle, foot progression angle, hip flexion, ab/adduction and internal/external rotation, knee flexion, ankle dorsiflexion, and ankle power were analyzed. A repeated-measures ANOVA followed by pairwise comparisons was used to analyze changes between three conditions. Toe-in running compared to normal and toe-out running reduced peak rearfoot eversion (mean difference (MD) with normal = 2.1°; p<0.001, MD with toe-out = 3.5°; p<0.001), peak pronation (MD with normal = -2.0°; p<0.001, MD with toe-out = -3.4; p = <0.001), and peak medial longitudinal arch angle (MD with normal = -0.7°; p = 0.022, MD with toe-out = -0.9; p = 0.005). Toe-out running significantly increased these kinematic factors compared to normal and toe-in running. Toe-in running compared to normal running increased peak hip internal rotation (MD = 2.3; p<0.001), and reduced peak knee flexion (MD = 1.3; p = 0.014). Toe-out running compared to normal running reduced peak hip internal rotation (MD = 2.5; p<0.001), peak hip ab/adduction (MD = 2.5; p<0.001), peak knee flexion (MD = 1.5; p = 0.003), peak ankle dorsiflexion (MD = 1.6; p<0.001), and peak ankle power (MD = 1.3; p = 0.001). Runners were able to change their foot progression angle when receiving real-time visual feedback for foot progression angle. Toe-in/toe-out running altered rearfoot kinematics and medial longitudinal arch angle, therefore supporting the potential value of gait retraining focused on foot progression angle using real-time visual feedback when atypical rearfoot in/eversion needs to be modified. It should be considered that changes in foot progression angle when running is accompanied by changes in lower limb joint biomechanics.

## Introduction

Running-related injuries (RRIs) are very common in athletes; sports clinicians are frequently consulted about these injuries [1]. Abnormal kinematics as intrinsic risk factors are considered to have an important role in the high incidence of RRIs [2]. Rearfoot eversion is among the most commonly reported kinematic factors in the studies investigating foot function and/or risk factors for lower-limb injuries [2–4]. As the subtalar coordination axis is not aligned with the foot coordination axes, and besides, no anatomical landmark exists on the talus, rearfoot eversion is predominantly measured as a surrogate to describe subtalar pronation [5, 6]. Much debate exists on whether atypical rearfoot eversion contributes to injury [4, 7]. This is mainly because most studies investigating rearfoot eversion for RRIs have either a case-control or a cross-sectional design that cannot prove causality; the results of prospective studies are mainly based on a small sample size [8]. There are several studies reporting that rearfoot eversion may be a potential risk factor for RRIs [2, 9, 10]. In a recent systematic review [2] we showed that peak rearfoot eversion may be associated with iliotibial band syndrome, patellar tendinopathy, and posterior tibial tendon dysfunction in runners. Female runners with atypical rearfoot eversion may be more prone to RRIs, specifically female runners with tibial stress fracture showed greater peak rearfoot eversion [11] and female runners with iliotibial band syndrome showed lower peak rearfoot eversion compared to non-injured runners [2, 12].

Atypical medial longitudinal arch angle (MLAA) is another contributing factor predisposing athletes to musculoskeletal overuse injuries [13]. MLAA collapse causes the calcaneus to evert in relation to the tibia, resulting in rearfoot eversion [14]. Normal rearfoot eversion and MLAA are essential for optimal shock absorption of the foot during the stance phase of gait. Atypical rearfoot eversion and MLAA may influence: 1. distribution of weight through the lower extremity, increasing force to the medial aspects of the foot and reducing shock absorption and postural balance abilities; and 2. alignment of the lower-limb kinematic chain, such as knee and hip mechanics [15]. For instance, excessive rearfoot eversion may result in excessive tibia internal rotation and hip internal rotation. This can result in faulty knee flexion/extension biomechanics which in turn produce a compensatory reaction in the tibiofemoral joint and may subsequently lead to patellofemoral symptoms [16]. Reduced shock absorption and malalignment are considered to play an important role in the development of running-related overuse injuries of the lower extremity [17].

In recent decades there has been increasing clinical and scientific interest in modifying atypical rearfoot eversion in order to prevent or manage RRI [18, 19]. External supports such as foot orthoses, motion control shoes, and therapeutic adhesive taping are the most common interventions studied to reduce excessive rearfoot eversion [20–22]. These are widely prescribed to realign or correct atypical rearfoot eversion. It is reported that foot orthoses may cause dependency and long-term negative psychological effects [23]. Most importantly, their effectiveness remains controversial [18]. So far, only a few studies have investigated the effect of a training program on excessive rearfoot eversion [24–28], showing sensory-motor training to be superior to either foot orthoses [26] or taping for correcting excessive rearfoot eversion [28]. More research into sports-related functional training interventions that modify rearfoot eversion is thus warranted.

Gait retraining is an increasingly used biomechanical modification intervention to practice a movement task [25, 29]. Gait retraining applying real-time feedback from an instrumented treadmill and/or motion capture through a projection and sound system is a novel way to induce motor strategies to alter movement patterns. Data obtained by tracking the biomechanics of the body are used to produce real-time feedback. Likewise, feedback modalities such as auditory and/or visual cues are provided for a training task to practice it at a given point/position. As these feedback modalities are given for any steps and individuals can perceive their own performance in the real time, this may help perform the training task more effectively than verbal feedback given by a coach or clinician. Step rate, step width, step length, foot strike, and hip adduction are the most common gait parameters on which virtual reality feedback is currently given to modify other running-related biomechanical risk factors [30–33]. These studies report successful findings for modifying biomechanical risk factors using the aforementioned parameters. Moreover, real-time visual feedback of kinematics or kinetics was reported as the most successful strategy to reduce high-risk factors for RRIs [31]. Therefore, gait retraining may be a useful approach to alter rearfoot biomechanics during running.

Previous studies show that rearfoot motion and MLAA might be influenced by changing foot progression angle (FPA) during running and walking [34–36]. In addition, FPA (foot abduction) is postulated as having a positive association with subtalar eversion because it is one of the subtalar pronation movement components [6, 37]. The assumption is that while running toe-out or toe-in, rearfoot kinematics are changed to more or less eversion, respectively. As inconsistent results have been reported about the effect/association of FPA on/with rearfoot eversion [34, 38–40], well-designed studies to investigate the effects of changing FPA on rearfoot kinematics are needed. Several studies have intervened FPA during walking using gait retraining to improve pain or reduce knee adduction moment, a contributing factor to knee osteoarthritis [41–43]. Nevertheless, it is unknown whether toe-in or toe-out positioning during running affects frontal plane rearfoot motion. Accordingly, it is postulated that running retraining with changing FPA may be useful to correct atypical rearfoot kinematics.

The main aim of this study is to investigate changes in rearfoot in/eversion while performing toe-in/toe-out running using real-time visual feedback. The secondary aim is to investigate changes in subtalar pronation/supination, MLAA, hip flexion, ab/adduction and internal/external rotation, knee flexion, ankle dorsiflexion, and ankle power while performing toe-out/in running. We hypothesized that toe-in running reduces peak rearfoot eversion, subtalar pronation, and MLAA, and toe-out running increases these factors relative to a natural FPA. From a clinical perspective, the findings might be helpful toward controlling atypical rearfoot kinematics when managing RRI.

## Methods

### Study design

This is a cross-sectional pilot study conducted to determine the feasibility and effects of changing FPA using real-time visual feedback on rearfoot in/eversion, subtalar supination/pronation, and MLAA.

### Setting

Data were collected at the Motion Lab of the Center for Rehabilitation, University Medical Center Groningen between January 2019 and April 2019.

### Participants

Seventeen female runners recruited by our advertisements and social media from the University of Groningen and local running clubs volunteered to participate. Inclusion criteria were: female, aged 18–40, minimum 1 year running experience, training distance >10 km/week, habitual rearfoot striker, free of self-reported lower-limb injuries or pain during the last 6 months, no musculoskeletal disorders and/or pain, no foot medial arch disorder determined using the navicular drop test, and no atypical static rearfoot in/eversion [44] prior to data collection. Two volunteers were excluded who had flat foot and/or excessive static rearfoot eversion. Fifteen volunteers who met the inclusion criteria participated in this study. Ethical approval was obtained through the local Medical Ethics Committee (METc 2018/086) of University Medical Center Groningen. Subjects signed an informed consent form and completed a self-developed questionnaire for demographic information prior to data collection.

### Instrumentation

Running assessments were performed on an instrumented split-belt treadmill with two integrated force plates of the Gait Real-time Analysis Interactive Lab (GRAIL) system (Motekforce Link, The Netherlands) [45]. Ground reaction force (GRF) signals were recorded at 1000Hz, combined with a 10-camera integrated motion capture system (Vicon Bonita 10; Vicon Motion Systems, Oxford, UK) and further processed to kinematic and kinetic variables in D-Flow (Version 3.28; Motekforce Link, The Netherlands) at a 100 Hz sampling frequency. Real-time filtering of the marker data was performed with a low-pass second-order zero phase Butterworth filter with a 6 Hz cut-off frequency.

### Marker placement

**Fig 1:**
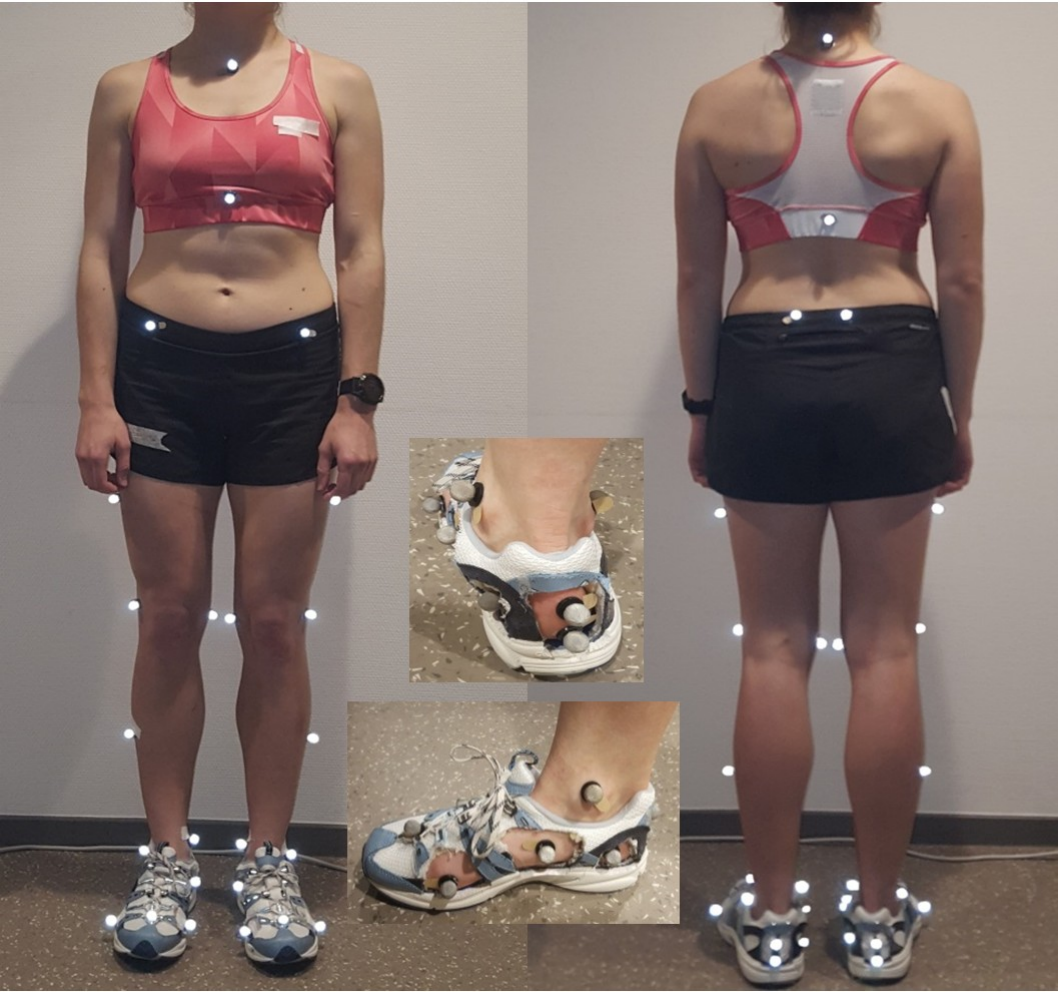
Marker placement. Twenty-six markers were attached to the body according to the HBM model; 8 markers were attached to the feet, to be used for calculating rearfoot eversion and MLAA.

Thirty-four markers were attached to the subject’s body by the same investigator (SHM). Of these, 26 markers were attached according to the human body model 2 (HBM2) (Fig 1) [29] and 8 markers were attached to both feet at the first metatarsal head, navicular bone tuberosity, medial side of calcaneus and posterior part of calcaneus for measuring rearfoot in/eversion and MLAA. Two markers attached along the vertical bisection of the heel counter and the marker on the medial aspect of the heel counter were considered for the rearfoot segment. Additionally, four holes were cut in the shoes: at the first metatarsal head, navicular bone tuberosity, medial side and posterior part of calcaneus (Fig 1). The holes were cut to uncover these aspects of the foot in order to attach markers directly to the skin, allowing measurement of foot movement and not shoe movement. All subjects wore the same brand of neutral shoes (Dr Comfort, refresh, USA) with the same neutral insole.

### Baseline measurement

To set a certain running speed for giving real-time feedback and generalize it to all runners, treadmill speed was set at 8 km/h for all conditions. After a 5-minute warm-up period, a 20-second baseline dataset was collected containing at least 20 strides. Baseline FPA was calculated using the average FPA in midstance (the value in 50% of stance phase) for the first 20 strides. FPA was considered the angle between the line connecting the markers on the calcaneus and second metatarsal head with the longitudinal axis of the treadmill.

### Feedback

**Fig 2:**
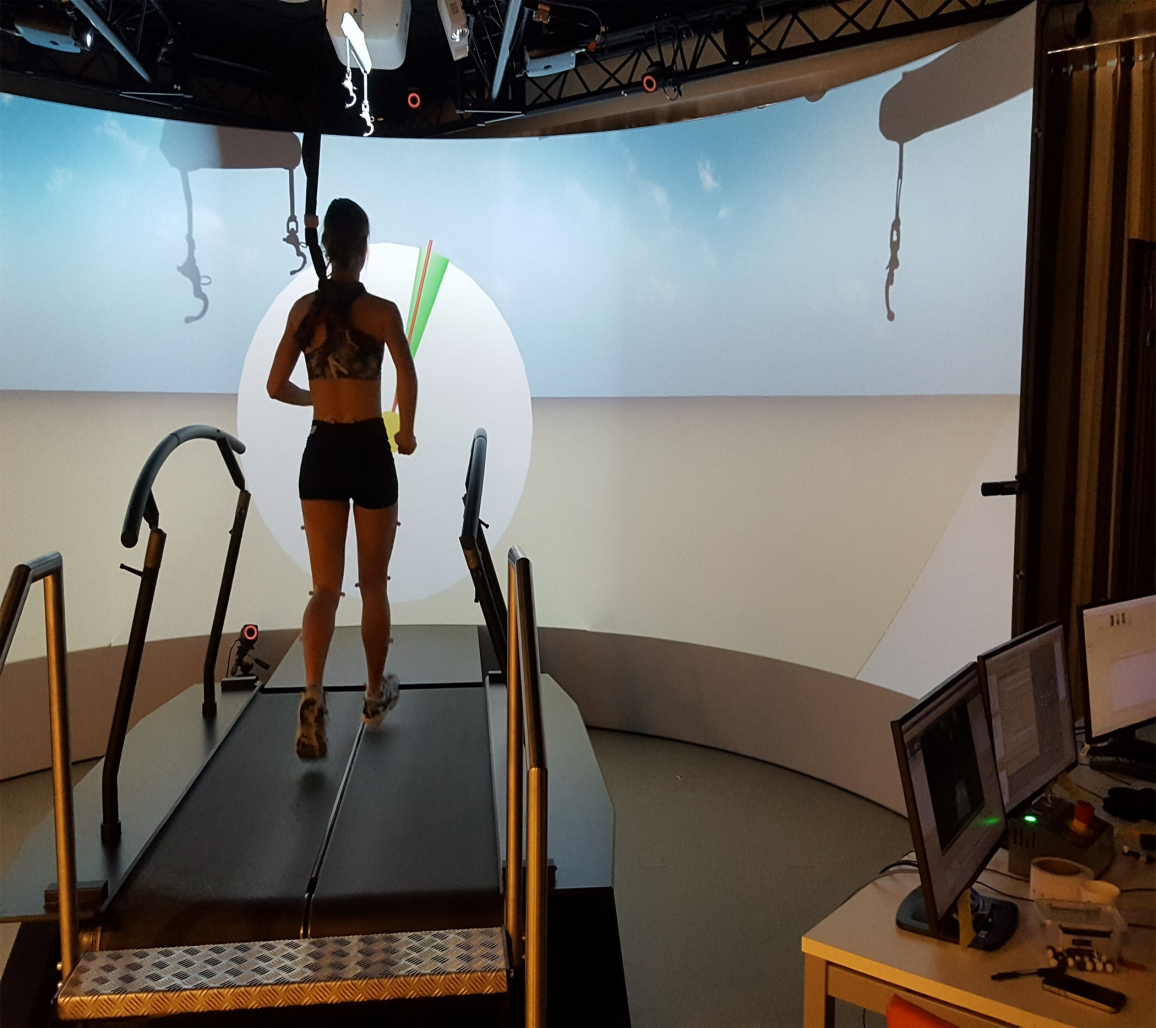
Picture representing real-time visual feedback for changing FPA. The training process: Real-time visual feedback is provided to the subject via the big screen. The red pointer represents the FPA of the right foot that is fixed in the midstance (50% stance phase) and updated on each step. The target area is a wedge with a 5° range, with its middle point specifying the subject’s normal FPA +5° for toe-out and -5° for toe-in. The aim is to turn the target area green (positive feedback) by keeping the red pointer (FPA) inside the target area. If the red pointer leaves the target area, the target area turns red (negative feedback).

A custom-made application was developed on the D-flow software to produce real-time feedback for toeing-out and toeing-in FPA. A clock with a red pointer set to FPA (degrees) was designed to reflect FPA during midstance in real time (Fig 2). A 5° target range was shown on the clock (green, Fig 2). To perform toeing-out FPA, the target range was set at 5° more than the baseline FPA average with an area of ±2.5° deviation from this point. Likewise, to perform toeing-in FPA, the target range was set at 5° less than the baseline FPA average with an area of ±2.5° deviation from this point. The 5° deviation from the baseline FPA was selected based on pilot testing. In the pilot testing, five runners were asked to run with various FPA (±5°, ±10°, and ±15°) relative to their baseline FPA. The 5° deviation from the baseline FPA (±5° FPA) was the only FPA that all runners ran with no difficulty. Prior to the feedback session, subjects were asked to practice the conditions with their dominant foot in the standing position to become familiar with the feedback. When the red pointer was located within the given target range, the area became green (positive feedback)–otherwise it became red (negative feedback). The red pointer was fixed on FPA in midstance and updated on each step. Before doing each task, subjects had a 2-min running with FPA feedback. An extra minute was allowed if needed. Subjects were then asked to run, and after 1-min running a 20-second dataset was collected. The order of the experimental tasks was randomized.

### Outcome measurement

The rearfoot segment coordinate system was established according to International Society of Biomechanics (ISB) recommendations [46] and calculated as rotation of local calcaneus coordination system relative to the fixed laboratory coordinate system using the rotation sequence defined by ISB. Subtalar pronation/supination angle was calculated using the Isman and Inman method [5], with the detailed explanation described in the study of van den Bogert et al. [29]. Because the pronation/supination axis is not aligned with the foot coordinate axes, it is defined in the foot reference frame using the average subtalar joint. MLAA was calculated based on the angle formed between three markers: first metatarsal head, navicular bone tuberosity and medial aspect of calcaneus. Hip, knee and ankle kinematics and ankle power are standard measures of HBM2 computed as explained by van den Bogert et al. [29]. GRF data were used to identify the stance phase, with a threshold of 10N vertical GRF for touchdown and toe-off. Kinematic and GRF data were filtered using low-pass zero phase second-order Butterworth filters with the same 6Hz cutoff frequency. Outcomes of ten consecutive steps following the given FPA were calculated and averaged within subjects before being averaged within conditions. Kinematic data during the stance phase were time-normalized to 100% of stance phase. Peak angles were explained as the maximum angle during the stance phase. Timing of peak angles was expressed as percentage of the stance phase. Angle excursions were expressed as range of motion from touchdown to peak angle. A custom MATLAB script (Version R2018a, Natick, MA, USA) was used to analyze data.

### Statistical analysis

Data were analyzed using IBM SPSS version 23 (IBM Corp., Armonk, NY, USA). The normality of data was checked by Shapiro-Wilk tests and QQ plots. A repeated-measures one-way ANOVA was performed for each outcome to identify statistically significant differences between conditions: baseline, toeing-out, and toeing-in trials. Significant main effects were followed up using pairwise comparisons with Bonferroni adjustment. Significance level (*α*) was set at 0.05.

## Results

**Fig 3:**
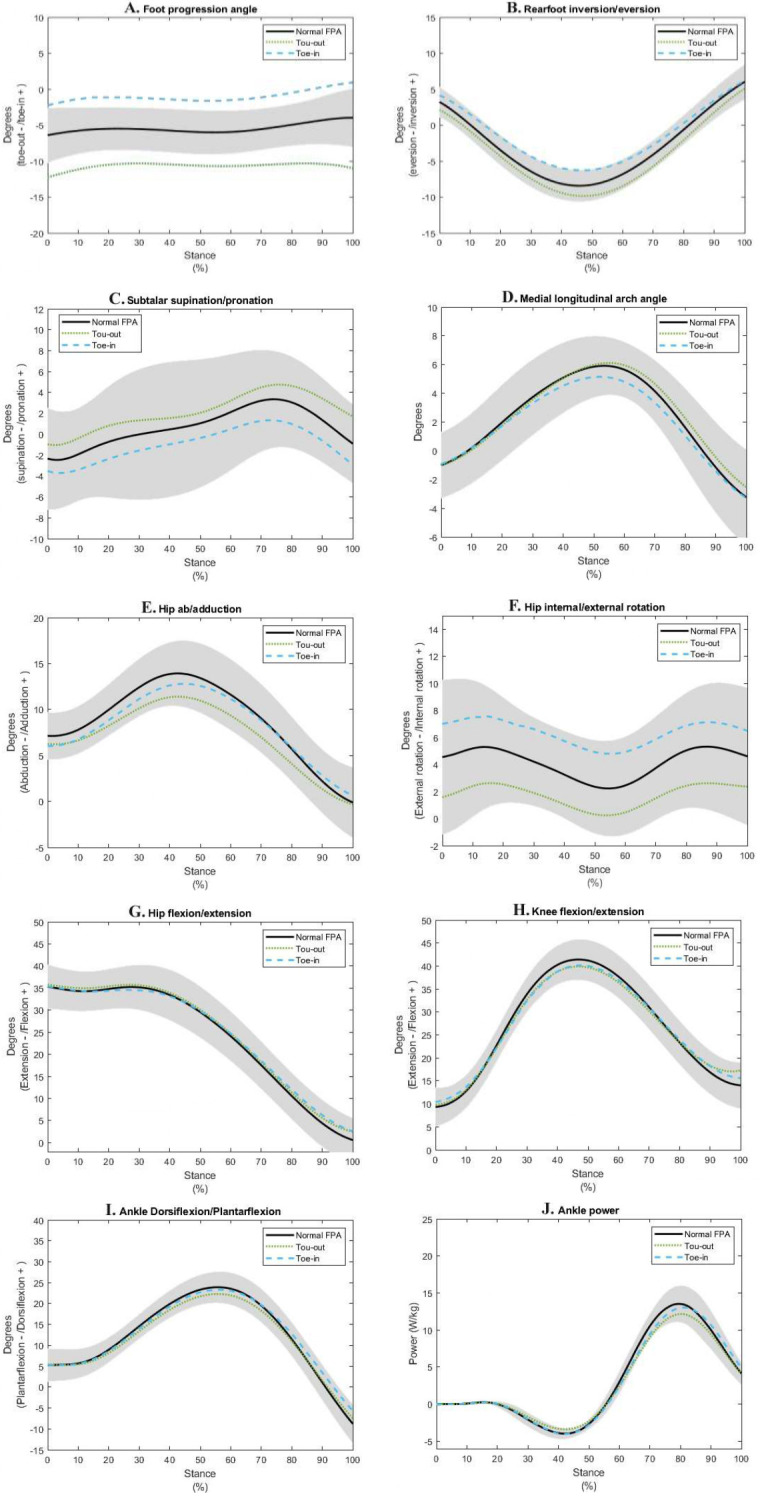
Ensemble average curves of measured variables for three FPA conditions, with 0% representing heel strike and 100% toe-off. Solid curves = normal FPA, dotted curves = toe-out condition, dashed curves = toe-in condition. Shaded area represents ±1 SD of the normal FPA condition.

Table 1 shows participants’ characteristics. All assumptions for repeated-measures ANOVA were met (no significant outliers, normal distribution, and sphericity). Table 2 shows results of one-way repeated-measures ANOVA analysis for all measured variables; Fig 3 shows the ensemble average curves of measured variables. The average FPA in midstance for normal, toe-out and toe-in running were -6.2°, -10.4°, and -1.8°, respectively; the average individual standard deviations were 1.1°, 1° and 0.9°, respectively.

**Table 1:** Participants characteristics.

Variable	Mean (SD)	Range
**Age, y**	27.5 (6.3)	21–40
**Height, cm**	170 (5)	164–182
**Weight, kg**	61.4 (6.1)	50–72
**Running experience, y**	6.3 (4.4)	2–17
**Weekly distance, km**	32.7 (17.4)	10–65

**Table 2:** Statistical results of the all variables^a^ for each FPA condition.

Variable	FPA condition			One-way repeated measures analysis		
	Normal FPA	Toe-out 5°	Toe-in 5°	F-value	P-value	Eta squared
*Foot progression angle (°)*	*-6*.*2 (3*.*1)*	*-10*.*4 (3*.*2)* *†	*-1*.*9 (3*.*2)* ‡	*444*.*66*	*< 0*.*001*	*0*.*97*
*Average individual SD for FPA(°)*	*1*.*1 (0*.*3)*	*1*.*0 (0*.*2)*	*0*.*9 (0*.*2)*	*1*.*77*	*0*.*189*	*0*.*11*
**Peak rearfoot eversion (°)**	-8.5 (2.2)	-9.9 (2.6) *†	-6.4 (2.2) ‡	103.16	**< 0.001**	0.88
Time to peak rearfoot eversion (% stance)	46.3 (2.7)	47.3 (3.0)	47.3 (3.7)	0.76	0.478	0.05
Rearfoot eversion at touchdown (°)	3.2 (2.1)	2.1 (2.1) *†	4.2 (2.3)	9.51	**0.001**	0.41
Rearfoot eversion excursion (°)	-11.5 (3.3)	-12.0 (3.6) †	-10.1 (3.2) ‡	20.46	**< 0.001**	0.59
**Peak pronation (°)**	4.4 (4.5)	5.8 (4.5) *†	2.4 (4.6) ‡	66.36	**< 0.001**	0.83
Time to peak pronation (% stance)	70.1 (15.2)	72.1 (17.3) †	69.6 (16)	4.56	**0.019**	0.25
Supination/pronation at touchdown (°)	-2.3 (4.9)	-1 (4.8) *†	-3.5 (4.9) ‡	24.69	**< 0.001**	0.64
Pronation excursion (°)	6.7 (4.2)	6.8 (3.4)	5.9 (4) ‡	3.57	**0.042**	0.20
**Peak MLAA (°)**	6.2 (2.2)	6.4 (2.2) †	5.5 (2.3) ‡	9.7	**0.001**	0.41
Time to peak MLAA (% stance)	54 (8.1)	56.6 (7.1) *	54.1 (9)	3.61	**0.040**	0.21
MLAA at touchdown (°)	-1.0 (2.3)	-1 (2.5)	-0.9 (2.7)	0.21	0.816	0.01
MLAA excursion (°)	7.2 (1.7)	7.4 (1.7) †	6.5 (1.7) ‡	13.02	**< 0.001**	0.48
**Peak hip internal rotation**	7.6 (4.8)	5.1 (5.2) *†	9.8 (5.1) ‡	71.28	**< 0.001**	0.84
Time to peak hip internal rotation (% stance)	58.3 (38.8)	59.9 (35.7)	51.9 (39.6)	1.70	0.214	0.11
Hip internal rotation at touchdown (°)	4.5 (5.7)	1.6 (5.9) *†	7.0 (5.8) ‡	82.25	**< 0.001**	0.86
Hip internal rotation excursion (°)	3.0 (2.7)	3.5 (2.5)	2.8 (3.0)	1.21	0.305	0.08
**Peak hip ab/adduction**	14.0 (3.6)	11.6 (3.4) *†	13.0 (4.6)	12.38	**< 0.001**	0.47
Time to peak hip ab/adduction (% stance)	43.2 (3.6)	43.4 (4.0)	45.7 (5.5)	4.02	0.055	0.22
Hip ab/adduction at touchdown (°)	7.1 (2.6)	6.3 (2.4)	6.0 (3.3)	3.06	0.063	0.18
Hip ab/adduction excursion (°)	6.9 (2.5)	5.3 (2.3) *†	7.0 (2.9)	28.45	**< 0.001**	0.67
**Peak hip flexion**	36.8 (5.5)	37.1 (5.6)	36.5 (5.2)	0.80	0.455	0.05
Time to peak hip flexion (% stance)	15.1 (16.0)	15.4 (16.3)	14.0 (16.9)	0.51	0.494	0.04
Hip flexion at touchdown (°)	35.3 (4.9)	35.8 (5.1)	35.4 (4.8)	0.62	0.546	0.04
Hip flexion excursion (°)	1.5 (2.2)	1.3 (1.9)	1.1 (1.6)	1.49	0.245	0.10
**Peak knee flexion**	41.5 (4.5)	40.0 (4.6) *	40.2 (4.9) ‡	8.81	**0.001**	0.39
Time to peak knee flexion (% stance)	48.1 (1.9)	48.1 (2.5)	48.6 (1.4)	0.83	0.445	0.06
Knee flexion at touchdown (°)	9.3 (4.2)	9.9 (4.2)	10.4 (3.8)	1.83	0.179	0.11
Knee flexion excursion (°)	32.1 (5.5)	30.2 (4.3) *	30.0 (4.7) ‡	11.46	**< 0.001**	0.45
**Peak ankle dorsiflexion**	24.0 (3.7)	22.4 (3.6) *†	23.4 (3.7)	10.30	**< 0.001**	0.42
Time to peak ankle dorsiflexion (% stance)	56.9 (2.1)	56.8 (2.5)	57.1 (2.3)	0.22	0.807	0.02
Ankle dorsiflexion at touchdown (°)	5.3 (3.9)	5.4 (2.9)	5.3 (3.7)	0.08	0.927	0.01
Ankle dorsiflexion excursion (°)	18.7 (2.5)	17.0 (2.7) *	18.0 (3.1)	5.50	**0.010**	0.28
**Peak ankle power**	13.7 (2.5)	12.3 (2.0) *†	13.3 (2.5)	11.7	**<0.001**	0.46

^a^ Values expressed as mean (SD), FPA foot progression angle, TD touchdown, MLAA medial longitudinal arch angle,

* significant difference between normal and toe-out FPA *p*<0.05,

† significant difference between toe-out and toe-in *p*<0.050,

‡ significant difference between normal and toe-in FPA *p*<0.05.

We found significant main effects of FPA conditions on peak rearfoot eversion (p<0.001), rearfoot eversion at touchdown (p = 0.001), and rearfoot eversion excursion (p<0.001). There was no significant main effect of FPA conditions on time to peak rearfoot eversion (p = 0.462). Post-hoc tests showed a significant difference in peak rearfoot eversion between normal and toe-out (mean difference (MD) = 1.4; p<0.001), between normal and toe-in (MD = -2.1; p<0.001), and between toe-out and toe-in (MD = -3.5; p<0.001). Post-hoc tests for rearfoot eversion excursion showed a significant difference between normal and toe-in (MD = -1.4; p = 0.001), and between toe-out and toe-in (MD = 1.9; p = 0.001). There was no significant difference in rearfoot eversion excursion between normal and toe-out (MD = -0.5; p = 0.1). Post-hoc tests for rearfoot eversion at touchdown showed significant differences between normal and toe-out (MD = 1.1; p = 0.037), and between toe-out and toe-in (MD = -2.0; p = 0.011).

We found significant main effects of FPA conditions on peak pronation (p<0.001), supination/pronation at touchdown (p<0.001), time to peak pronation (p = 0.019), and pronation excursion (p = 0.042). Pairwise comparisons showed a significant difference in peak pronation between normal and toe-out (MD = -1.4; p = 0.002), between normal and toe-in (MD = 2; p<0.001), and between toe-out and toe-in (MD = 3.4; p<0.001). Post-hoc tests for pronation at touchdown showed a significant difference between normal and toe-out (MD = -1.4; p = 0.015), between normal and toe-in (MD = 1.2; p = 0.004), and between toe-out and toe-in (MD = 2.6; p<0.001). Post-hoc tests showed a significant difference in time to peak pronation between toe-out and toe-in (MD = 2.5; p = 0.035). There were no significant differences in time to peak pronation between normal and toe-in (MD = 0.5; p = 0.999) and between normal and toe-out (MD = -2.1; p = 0.219).

We found significant main effects of FPA conditions on peak MLAA (p = 0.001), time to peak MLAA (p = 0.04), and MLAA excursion (p<0.001). There was no significant main effect of FPA conditions on MLAA at touchdown (p = 0.816). Bonferroni post-hoc tests showed a significant difference in peak MLAA between normal and toe-in (MD = 0.7; p = 0.022), and between toe-out and toe-in (MD = 0.9; p = 0.005). There was no significant difference in peak MLAA between normal and toe-out (MD = -0.2; p = 0.876). Post-hoc tests also showed a significant difference in time to peak MLAA between normal and toe-out (MD = -2.6; p = 0.033) and a significant difference in MLAA excursion between normal and toe-in (MD = 0.8; p = 0.005), and between toe-out and toe-in (MD = 1; p<0.001).

We found significant main effects of FPA conditions on peak hip internal/external rotation (p<0.001), and hip internal/external rotation at touchdown (p<0.001). There were no significant main effects of FPA conditions on time to peak hip internal/external rotation (p = 0.214), and hip internal/external rotation excursion (p = 0.305). Post-hoc tests showed a significant difference in peak hip internal/external rotation between normal and toe-out (MD = 2.5; p<0.001), between toe-out and toe-in (MD = -4.8; p<0.001), and between normal and toe-in (MD = -2.3; p<0.001). Post-hoc tests also showed a significant difference in hip internal/external rotation at touchdown between normal and toe-out (MD = 3.0; p<0.001), between toe-out and toe-in (MD = -5.4; p<0.001), and between normal and toe-in (MD = -2.5; p<0.001).

We found significant main effects of FPA conditions on peak hip ab/adduction (p<0.001), and hip ab/adduction excursion (p<0.001). There were no significant main effects of FPA conditions on hip ab/adduction at touchdown (p = 0.063), and time to peak hip ab/adduction (p = 0.055). Post-hoc tests showed a significant difference in peak hip ab/adduction between normal and toe-out (MD = 2.5; p<0.001), and between toe-out and toe-in (MD = -1.4; p = 0.044). There was no significant difference in peak hip ab/adduction between normal and toe-in (MD = 1.0; p = 0.292). Post-hoc tests also showed a significant difference in hip ab/adduction excursion between normal and toe-out (MD = 1.6; p<0.001), and between toe-out and toe-in (MD = -1.7; p = 0.044). There was no significant difference in hip ab/adduction excursion between normal and toe-in (MD = -0.1; p = 0.999).

We found no significant main effects of FPA conditions on peak hip flexion (p = 0.455), time to peak hip flexion (p = 0.494), hip flexion at touchdown (p = 0.546), and hip flexion excursion (p = 0.245).

We found significant main effects of FPA conditions on peak knee flexion (p = 0.001), and knee flexion excursion (p<0.001). There were no significant main effects of FPA conditions on time to peak knee flexion (p = 0.445), and knee flexion at touchdown (p = 0.179). Post-hoc tests showed a significant difference in peak knee flexion between normal and toe-out (MD = 1.5; p = 0.003), and between normal and toe-in (MD = 1.3; p = 0.014). There was no significant difference in peak knee flexion between toe-out and toe-in (MD = -0.2; p = 0.999). Post-hoc tests showed a significant difference in knee flexion excursion between normal and toe-out (MD = 2.0; p = 0.003), and between normal and toe-in (MD = 2.4; p = 0.005). There was no significant difference in knee flexion excursion between toe-out and toe-in (MD = 0.4; p = 0.999).

We found significant main effects of FPA conditions on peak ankle dorsiflexion (p<0.001), and ankle dorsiflexion excursion (p = 0.010). There were no significant main effects of FPA conditions on time to peak ankle dorsiflexion (p = 0.807), and ankle dorsiflexion at touchdown (p = 0.927). Post-hoc tests showed a significant difference in peak ankle dorsiflexion between normal and toe-out (MD = 1.6; p<0.001), and between toe-out and toe-in (MD = -1.0; p = 0.011). There was no significant difference in peak ankle dorsiflexion between normal and toe-in (MD = 0.6; p = 0.653). Post-hoc tests also showed a significant difference in ankle dorsiflexion excursion between normal and toe-out (MD = 1.8; p = 0.001). There was no significant difference in ankle dorsiflexion excursion between normal and toe-in (MD = 0.6; p = 0.916), and between toe-out and toe-in (MD = -1.1; p = 0.258).

We found significant main effects of FPA conditions on peak ankle power (p<0.001). Post-hoc tests showed a significant difference in peak ankle power between normal and toe-out (MD = 1.3; p = 0.001), and between toe-out and toe-in (MD = -0.9; p<0.005). There was no significant difference in peak ankle power between normal and toe-in (MD = 0.4; p = 590).

## Discussion

The main aim of this study was to investigate the immediate effects of toe-in/toe-out running using real-time visual feedback on rearfoot in/eversion, subtalar pronation/supination, and MLAA during running. In support of our hypothesis, peak rearfoot eversion, peak subtalar pronation and peak MLAA were reduced by toe-in running and increased by toe-out running compared to normal running. Additionally, toe-in running reduced rearfoot eversion excursion, MLAA excursion, and subtalar pronation at touchdown compared to normal and toe-out running. Nowadays gait retraining is increasingly used in clinical practice to prevent and manage a variety of sports injuries. Since modifying atypical rearfoot in/eversion is of great interest to biomechanical research and clinical practices, our study constitutes a feasible and applicable basis for using real-time visual feedback to perform toe-in/toe-out running in order to change rearfoot in/eversion, subtalar supination/pronation, and MLAA.

No study has so far investigated the kinematic effects of changing FPA using real-time visual feedback during running. We considered ±5° differences from the subject’s normal FPA as target points. Participants generally responded in accordance with the target points. The target area on the clock was set ±2.5° from the target point for both toe-in and toe-out conditions. The results of the averaged individual SDs show that participants successfully changed their FPA based on the target area (2SD = 2 for toe-out and 1.8 for toe-in). None reported any problems with changing the FPA when asked about any difficulties during performing tasks. The average change of FPA relative to normal FPA was 4.2° for toe-out running and 4.4° for toe-in running. In fact, the FPA display in the real-time feedback during familiarization helped subjects adapt to the experiment. Because the pointer was aligned with the subject’s FPA, it could be easily perceived.

We hypothesized that toe-in running reduces peak rearfoot eversion and toe-out running increases peak rearfoot eversion. Excessive rearfoot eversion is a modifiable risk factor for overuse injuries in athletes [47]. We showed that moving from toe-out to toe-in resulted in a reduction in peak eversion and subtalar pronation, thus supporting the potential value of changing FPA as a method for gait retraining in order to modify atypical rearfoot in/eversion. These alterations in peak rearfoot in/eversion by toe-in/toe-out running might be partially due to lateral and medial shifting of foot pressure during foot roll-over by toe-in and toe-out, respectively [48]. Our results showed that toe-in running reduces peak rearfoot eversion by 2.1° –a promising result, as a recent systematic review and meta-analysis investigating rearfoot eversion in injured runners and controls showed that a 2° increase in peak rearfoot eversion distinguishes injured from healthy runners [2]. Hence toe-in/toe-out running with a 4–5° difference from the preferred FPA may have clinical significance in the control of atypical rearfoot in/eversion when preventing and managing RRI.

Subtalar pronation at touchdown and time to peak pronation were significantly reduced by toe-in running relative to normal running; however, rearfoot eversion at touchdown and time to peak rearfoot eversion were not significant between different FPA conditions. Rearfoot eversion excursion was also reduced during toe-in running compared to normal and toe-out running. Supination and/or inversion are directly attributed to tarsal joint locking in either the early or the late stance phase [49]. Accordingly, supination helps the foot turn to a rigid lever where needed. It is documented that greater pronation excursion leads to a delayed supination in the late stance [50]. According to our results, in toe-in running the foot is in a more supinated position relative to normal and toe-out running. Therefore, in individuals who have greater foot pronation, toe-running may help foot stabilization at touchdown and even in late stance phase during running.

We found a smaller MLAA during toe-in running compared with normal and toe-out running. The effect of changing FPA on MLAA during running is not yet well documented. Only few studies with conflicting results investigated the association between MLAA and FPA during running or walking [36, 51, 52], examining only the correlation between MLAA and FPA but not how changeable MLAA is when changing FPA. We found a decreased MLAA of approximately 0.7° and 1° during toe-in running compared to normal and toe-out running, respectively. This amount of change might appear small compared to changes possibly required to correct an atypical MLAA. An explanation for a small change in MLAA might be that the current study was conducted for effects on healthy subjects in order to assess the potential of FPA modification on MLAA. Further research on individuals with atypical MLAA is therefore warranted to determine how effective FPA modifications are on MLAA. This is even more important because the correction of atypical MLAA has often been suggested as one of the potential corrective strategies for atypical rearfoot eversion [50, 53].

Our results showed that changes in FPA are accompanied by changes in lower limb joint biomechanics. Specifically, these changes occurred in the peak hip internal/external rotation, hip ab/adduction, knee flexion, ankle dorsiflexion, and ankle power. Compared to running with normal FPA, both toe-out and toe-in running reduced peak hip adduction. As increased peak hip adduction is associated with iliotibial band syndrome and patellofemoral pain syndrome in runners [12, 54], FPA modification might be used as a potential gait retraining to reduce peak hip adduction. Toe-out running was accompanied by increased hip external rotation which is reported as a risk factor for medial tibial stress syndrome [55]. In contrast, toe-in running was accompanied by increased hip internal rotation. Toe-in running, therefore, may be used as potential gait retraining to reduce increased hip external rotation. Toe-out running compared to running with normal FPA reduced ankle power, possibly resulting in reducing the effectiveness of the ankle in providing positive push-off power. Gait retraining studies have shown that the peak knee adduction moment, a contributing factor to knee osteoarthritis, is increased/decreased with changes in FPA [41, 56]. This can subsequently load different aspects of the knee. As a result, clinician and researchers should consider changes in lower limb joint biomechanics when using FPA to modify rearfoot eversion.

Rearfoot in/eversion has been commonly used in the literature as an alternative way to express subtalar supination/pronation during walking or running [4, 6]. Our results show that although peak rearfoot eversion can be a proper representative of peak subtalar pronation during running, there are considerable differences between the other variables such as angle at touchdown, time to peak and excursion. Rearfoot eversion only describes one aspect of subtalar pronation and the other aspects of subtalar pronation may distinguish it from rearfoot eversion during running. Isman and Inman [5] presented a 3D kinematic approach defining true supination/pronation angle occurring in the subtalar joint as used in the current study. It is therefore suggested that future studies apply proper terminology (supination/pronation and/or rearfoot in/eversion) based on the study objective.

One major concern about rearfoot eversion is that no standardized norm or clinical definition exists for classification of rearfoot eversion during running to specify the extent to which rearfoot eversion falls into typical or atypical movements [4]. In the current study, the use of the terms typical or atypical (greater or lower) rearfoot eversion during running is based on the results of studies investigating rearfoot eversion between injured (history of injury) and non-injured runners. Therefore, further studies warrant to determine to what extent rearfoot eversion can be considered as typical or atypical.

The current study showed the feasibility and effects of toe-in/toe-out running using real-time visual feedback on rearfoot in/eversion, subtalar pronation/supination, and MLAA. To successfully achieve a target FPA during running an advanced system is needed, as running is a fast-cyclic motion that makes real-time feedback difficult. Current approaches for giving feedback on FPA mainly require camera-based motion capture, limiting FPA measurement or/and training to laboratory settings (thus hindering FPA training outside the laboratory). Recent studies present valid means using insole- or shoe-embedded sensors to estimate FPA and give real-time feedback during over-ground gait [57, 58]. However, these means were validated during walking only, therefore usability investigations during running are required. Also, compared to the motion capture values an absolute error of 1.7° should be taken into account when using these means. To see the effectiveness of gait retraining on rearfoot eversion over time or to be sure that change in rearfoot eversion is at the desired level, tracking rearfoot eversion might be useful. As application of 3D motion capture systems is not easy in clinical practice, 2D measurement of rearfoot eversion using smartphone application can be used as a surrogate to 3D measurement [59].

### Limitations and recommendations

There are a few limitations in the current study. All participants were healthy female runners so results cannot be extrapolated to male runners and/or injured runners. Further research is needed to investigate whether our results have the same effect on runners with atypical in/eversion, supinated or pronated feet, and/or MLAA. All participants ran with rearfoot strike. Since foot strike pattern affects the ankle and foot biomechanics, our results cannot be generalized to those with midfoot or forefoot strike. Based on our results regarding the differences between rearfoot eversion and subtalar pronation it was suggested that future research conducted to investigate the biomechanics of subtalar pronation in individuals should consider the difference between rearfoot in/eversion and subtalar supination/pronation, and choose one or both based on the study objective. Running speed was set at 8km/h, so it is not clear whether the same results would be found at higher or slower speeds. Changes in lower limb biomechanics such as hip ab/adduction, hip internal/external rotation, knee flexion, ankle dorsiflexion, and ankle power should be taken into account when changing FPA is used to modify rearfoot eversion. As our study aimed to investigate the acute effect of changing FPA, it is unknown whether runners would retain it in the long term. Therefore, further studies should be undertaken to investigate the viability of toe-in/toe-out running in the long term.

## Conclusion

This study showed that female healthy runners were able to change their FPA when receiving real-time visual feedback for FPA. Toe-in running using real-time visual feedback reduced peak rearfoot eversion, peak pronation, and peak MLAA compared to normal and toe-out running. Toe-out running, instead, increased these kinematic factors compared to normal and toe-in running. Rearfoot in/eversion is not an appropriate surrogate to predict all supination/pronation parameters. Our study provides new knowledge and lays the foundation for future research into modifying atypical rearfoot in/eversion, subtalar supination/pronation, and MLAA using gait retraining (toe-in and toe-out running) by real-time visual feedback during running. Clinicians and researchers should take it into account that changes in FPA when running is accompanied by changes in lower limb joint biomechanics.

## Supporting information

S1 Fig One-way repeated measure ANOVA results for foot progression angle (FPA). (DOCX)

S2 Fig One-way repeated measure ANOVA results for rearfoot eversion variables. (DOCX)

S3 Fig One-way repeated measure ANOVA results for pronation variables. (DOCX)

S4 Fig One-way repeated measure ANOVA results for medial longitudinal arch angle. (DOCX)

S5 Fig One-way repeated measure ANOVA results for hip internal rotation. (DOCX)

S6 Fig One-way repeated measure ANOVA results for hip ab/adduction. (DOCX)

S7 Fig One-way repeated measure ANOVA results for hip flexion. (DOCX)

S8 Fig One-way repeated measure ANOVA results for knee flexion. (DOCX)

S9 Fig One-way repeated measure ANOVA results for ankle dorsiflexion. (DOCX)

S10 Fig One-way repeated measure ANOVA results for peak ankle power. (DOCX)
