# Supplementary material for: The effect of changing foot progression angle using real-time visual feedback on rearfoot eversion during running
Source: PLoS One. 2021 Feb 10;16(2):e0246425. doi: 10.1371/journal.pone.0246425 (PMC7875396; doi:10.1371/journal.pone.0246425)
Supplement: S1 Fig — (DOCX) [file pone.0246425.s001.docx]

**S1 Fig**. One-way repeated measure ANOVA results for foot progression angle (FPA)

| **Within-Subjects Factors** | |
| --- | --- |
| Measure: MEASURE_1 | |
| FPA | Dependent Variable |
| 1 | FPAbase |
| 2 | FPAplus |
| 3 | FPAminus |

| **Descriptive Statistics** | | | |
| --- | --- | --- | --- |
|  | Mean | Std. Deviation | N |
| FPAbase | -6,230 | 3,1590 | 15 |
| FPAplus | -10,363 | 3,6575 | 15 |
| FPAminus | -1,796 | 3,1211 | 15 |

| **Tests of Within-Subjects Effects** | | | | | | | |
| --- | --- | --- | --- | --- | --- | --- | --- |
| Measure: MEASURE_1 | | | | | | | |
| Source | | Type III Sum of Squares | df | Mean Square | F | Sig. | Partial Eta Squared |
| FPA | Sphericity Assumed | 550,707 | 2 | 275,353 | 118,531 | ,000 | ,894 |
|  | Greenhouse-Geisser | 550,707 | 1,483 | 371,401 | 118,531 | ,000 | ,894 |
|  | Huynh-Feldt | 550,707 | 1,617 | 340,550 | 118,531 | ,000 | ,894 |
|  | Lower-bound | 550,707 | 1,000 | 550,707 | 118,531 | ,000 | ,894 |
| Error(FPA) | Sphericity Assumed | 65,046 | 28 | 2,323 |  |  |  |
|  | Greenhouse-Geisser | 65,046 | 20,759 | 3,133 |  |  |  |
|  | Huynh-Feldt | 65,046 | 22,640 | 2,873 |  |  |  |
|  | Lower-bound | 65,046 | 14,000 | 4,646 |  |  |  |

| **Pairwise Comparisons** | | | | | | |
| --- | --- | --- | --- | --- | --- | --- |
| Measure: MEASURE_1 | | | | | | |
| (I) FPA | (J) FPA | Mean Difference (I-J) | Std. Error | Sig.^b^ | 95% Confidence Interval for Difference^b^ | |
|  |  |  |  |  | Lower Bound | Upper Bound |
| 1 | 2 | 4,233^*^ | ,692 | ,000 | 2,251 | 6,014 |
|  | 3 | -4,435^*^ | ,407 | ,000 | -5,541 | -3,328 |
| 2 | 1 | -4,233^*^ | ,692 | ,000 | -6,014 | -2,251 |
|  | 3 | -8,567^*^ | ,533 | ,000 | -10,016 | -7,119 |
| 3 | 1 | 4,435^*^ | ,407 | ,000 | 3,328 | 5,541 |
|  | 2 | 8,567^*^ | ,533 | ,000 | 7,119 | 10,016 |
| Based on estimated marginal means | | | | | | |
| *. The mean difference is significant at the ,05 level. | | | | | | |
| b. Adjustment for multiple comparisons: Bonferroni. | | | | | | |
