# Supplementary material for: The effect of changing foot progression angle using real-time visual feedback on rearfoot eversion during running
Source: PLoS One. 2021 Feb 10;16(2):e0246425. doi: 10.1371/journal.pone.0246425 (PMC7875396; doi:10.1371/journal.pone.0246425)
Supplement: S2 Fig — (DOCX) [file pone.0246425.s002.docx]

**S2 Fig**. One-way repeated measure ANOVA results for rearfoot eversion variables

**A: peak rearfoot eversion**

| **Within-Subjects Factors** | |
| --- | --- |
| Measure: MEASURE_1 | |
| FPA | Dependent Variable |
| 1 | Rearfootbase_peak |
| 2 | Rearfootplus_peak |
| 3 | Rearfootminus_peak |

| **Descriptive Statistics** | | | |
| --- | --- | --- | --- |
|  | Mean | Std. Deviation | N |
| Rearfootbase_peak | 8,476 | 2,2053 | 15 |
| Rearfootplus_peak | 9,887 | 2,6189 | 15 |
| Rearfootminus_peak | 6,354 | 2,1893 | 15 |

| **Tests of Within-Subjects Effects** | | | | | | | | | |
| --- | --- | --- | --- | --- | --- | --- | --- | --- | --- |
| Measure: MEASURE_1 | | | | | | | | | |
| Source | | Type III Sum of Squares | df | Mean Square | F | Sig. | Partial Eta Squared | Noncent. Parameter | Observed Power^a^ |
| FPA | Sphericity Assumed | 94,886 | 2 | 47,443 | 103,162 | ,000 | ,881 | 206,324 | 1,000 |
|  | Greenhouse-Geisser | 94,886 | 1,616 | 58,702 | 103,162 | ,000 | ,881 | 166,751 | 1,000 |
|  | Huynh-Feldt | 94,886 | 1,796 | 52,820 | 103,162 | ,000 | ,881 | 185,320 | 1,000 |
|  | Lower-bound | 94,886 | 1,000 | 94,886 | 103,162 | ,000 | ,881 | 103,162 | 1,000 |
| Error(FPA) | Sphericity Assumed | 12,877 | 28 | ,460 |  |  |  |  |  |
|  | Greenhouse-Geisser | 12,877 | 22,629 | ,569 |  |  |  |  |  |
|  | Huynh-Feldt | 12,877 | 25,150 | ,512 |  |  |  |  |  |
|  | Lower-bound | 12,877 | 14,000 | ,920 |  |  |  |  |  |
| 1. Computed using alpha = ,05 | | | | | | | | | |

| **Pairwise Comparisons** | | | | | | |
| --- | --- | --- | --- | --- | --- | --- |
| Measure: MEASURE_1 | | | | | | |
| (I) FPA | (J) FPA | Mean Difference (I-J) | Std. Error | Sig.^b^ | 95% Confidence Interval for Difference^b^ | |
|  |  |  |  |  | Lower Bound | Upper Bound |
| 1 | 2 | -1,411^*^ | ,230 | ,000 | -2,035 | -,787 |
|  | 3 | 2,122^*^ | ,202 | ,000 | 1,573 | 2,671 |
| 2 | 1 | 1,411^*^ | ,230 | ,000 | ,787 | 2,035 |
|  | 3 | 3,533^*^ | ,301 | ,000 | 2,716 | 4,350 |
| 3 | 1 | -2,122^*^ | ,202 | ,000 | -2,671 | -1,573 |
|  | 2 | -3,533^*^ | ,301 | ,000 | -4,350 | -2,716 |
| Based on estimated marginal means | | | | | | |
| *. The mean difference is significant at the ,05 level. | | | | | | |
| b. Adjustment for multiple comparisons: Bonferroni. | | | | | | |

**B. Time to peak rearfoot eversion**

| **Within-Subjects Factors** | |
| --- | --- |
| Measure: MEASURE_1 | |
| FPA | Dependent Variable |
| 1 | Rearbase_time |
| 2 | Rearplus_time |
| 3 | Rearminus_time |

| **Descriptive Statistics** | | | |
| --- | --- | --- | --- |
|  | Mean | Std. Deviation | N |
| Rearbase_time | 46,27 | 2,658 | 15 |
| Rearplus_time | 47,33 | 2,992 | 15 |
| Rearminus_time | 47,33 | 3,754 | 15 |

| **Tests of Within-Subjects Effects** | | | | | | | |
| --- | --- | --- | --- | --- | --- | --- | --- |
| Measure: MEASURE_1 | | | | | | | |
| Source | | Type III Sum of Squares | df | Mean Square | F | Sig. | Partial Eta Squared |
| FPA | Sphericity Assumed | 11,378 | 2 | 5,689 | ,759 | ,478 | ,051 |
|  | Greenhouse-Geisser | 11,378 | 1,454 | 7,827 | ,759 | ,441 | ,051 |
|  | Huynh-Feldt | 11,378 | 1,579 | 7,207 | ,759 | ,451 | ,051 |
|  | Lower-bound | 11,378 | 1,000 | 11,378 | ,759 | ,398 | ,051 |
| Error(FPA) | Sphericity Assumed | 209,956 | 28 | 7,498 |  |  |  |
|  | Greenhouse-Geisser | 209,956 | 20,352 | 10,316 |  |  |  |
|  | Huynh-Feldt | 209,956 | 22,101 | 9,500 |  |  |  |
|  | Lower-bound | 209,956 | 14,000 | 14,997 |  |  |  |

| **Pairwise Comparisons** | | | | | | |
| --- | --- | --- | --- | --- | --- | --- |
| Measure: MEASURE_1 | | | | | | |
| (I) FPA | (J) FPA | Mean Difference (I-J) | Std. Error | Sig.^a^ | 95% Confidence Interval for Difference^a^ | |
|  |  |  |  |  | Lower Bound | Upper Bound |
| 1 | 2 | -1,067 | ,636 | ,347 | -2,795 | ,662 |
|  | 3 | -1,067 | 1,193 | 1,000 | -4,309 | 2,176 |
| 2 | 1 | 1,067 | ,636 | ,347 | -,662 | 2,795 |
|  | 3 | ,000 | 1,082 | 1,000 | -2,941 | 2,941 |
| 3 | 1 | 1,067 | 1,193 | 1,000 | -2,176 | 4,309 |
|  | 2 | ,000 | 1,082 | 1,000 | -2,941 | 2,941 |
| Based on estimated marginal means | | | | | | |
| a. Adjustment for multiple comparisons: Bonferroni. | | | | | | |

**C: Rearfoot eversion at touchdown**

| **Within-Subjects Factors** | |
| --- | --- |
| Measure: MEASURE_1 | |
| FPA | Dependent Variable |
| 1 | Rearfootbase_TD |
| 2 | Rearfootplus_TD |
| 3 | rearfootminus_TD |

| **Descriptive Statistics** | | | |
| --- | --- | --- | --- |
|  | Mean | Std. Deviation | N |
| Rearfootbase_TD | -3,219 | 2,0951 | 15 |
| Rearfootplus_TD | -2,123 | 2,0818 | 15 |
| rearfootminus_TD | -4,155 | 2,3311 | 15 |

| **Tests of Within-Subjects Effects** | | | | | | | |
| --- | --- | --- | --- | --- | --- | --- | --- |
| Measure: MEASURE_1 | | | | | | | |
| Source | | Type III Sum of Squares | df | Mean Square | F | Sig. | Partial Eta Squared |
| FPA | Sphericity Assumed | 31,020 | 2 | 15,510 | 9,510 | ,001 | ,405 |
|  | Greenhouse-Geisser | 31,020 | 1,491 | 20,802 | 9,510 | ,002 | ,405 |
|  | Huynh-Feldt | 31,020 | 1,628 | 19,051 | 9,510 | ,002 | ,405 |
|  | Lower-bound | 31,020 | 1,000 | 31,020 | 9,510 | ,008 | ,405 |
| Error(FPA) | Sphericity Assumed | 45,667 | 28 | 1,631 |  |  |  |
|  | Greenhouse-Geisser | 45,667 | 20,877 | 2,187 |  |  |  |
|  | Huynh-Feldt | 45,667 | 22,796 | 2,003 |  |  |  |
|  | Lower-bound | 45,667 | 14,000 | 3,262 |  |  |  |

| **Pairwise Comparisons** | | | | | | |
| --- | --- | --- | --- | --- | --- | --- |
| Measure: MEASURE_1 | | | | | | |
| (I) FPA | (J) FPA | Mean Difference (I-J) | Std. Error | Sig.^b^ | 95% Confidence Interval for Difference^b^ | |
|  |  |  |  |  | Lower Bound | Upper Bound |
| 1 | 2 | -1,096^*^ | ,383 | ,037 | -2,136 | -,056 |
|  | 3 | ,936 | ,402 | ,107 | -,158 | 2,029 |
| 2 | 1 | 1,096^*^ | ,383 | ,037 | ,056 | 2,136 |
|  | 3 | 2,032^*^ | ,587 | ,011 | ,437 | 3,626 |
| 3 | 1 | -,936 | ,402 | ,107 | -2,029 | ,158 |
|  | 2 | -2,032^*^ | ,587 | ,011 | -3,626 | -,437 |
| Based on estimated marginal means | | | | | | |
| *. The mean difference is significant at the ,05 level. | | | | | | |
| b. Adjustment for multiple comparisons: Bonferroni. | | | | | | |

**D: Rearfoot eversion Excursion**

| **Within-Subjects Factors** | |
| --- | --- |
| Measure: MEASURE_1 | |
| FPA | Dependent Variable |
| 1 | Rearbase_excurs |
| 2 | Rearplus_excurs |
| 3 | Rearminus_excurs |

| **Descriptive Statistics** | | | |
| --- | --- | --- | --- |
|  | Mean | Std. Deviation | N |
| Rearbase_excurs | 11,479 | 3,3013 | 15 |
| Rearplus_excurs | 12,014 | 3,6330 | 15 |
| Rearminus_excurs | 10,093 | 3,2409 | 15 |

| **Tests of Within-Subjects Effects** | | | | | | | |
| --- | --- | --- | --- | --- | --- | --- | --- |
| Measure: MEASURE_1 | | | | | | | |
| Source | | Type III Sum of Squares | df | Mean Square | F | Sig. | Partial Eta Squared |
| FPA | Sphericity Assumed | 29,503 | 2 | 14,751 | 20,456 | ,000 | ,594 |
|  | Greenhouse-Geisser | 29,503 | 1,473 | 20,023 | 20,456 | ,000 | ,594 |
|  | Huynh-Feldt | 29,503 | 1,605 | 18,385 | 20,456 | ,000 | ,594 |
|  | Lower-bound | 29,503 | 1,000 | 29,503 | 20,456 | ,000 | ,594 |
| Error(FPA) | Sphericity Assumed | 20,191 | 28 | ,721 |  |  |  |
|  | Greenhouse-Geisser | 20,191 | 20,628 | ,979 |  |  |  |
|  | Huynh-Feldt | 20,191 | 22,465 | ,899 |  |  |  |
|  | Lower-bound | 20,191 | 14,000 | 1,442 |  |  |  |

| **Pairwise Comparisons** | | | | | | |
| --- | --- | --- | --- | --- | --- | --- |
| Measure: MEASURE_1 | | | | | | |
| (I) FPA | (J) FPA | Mean Difference (I-J) | Std. Error | Sig.^b^ | 95% Confidence Interval for Difference^b^ | |
|  |  |  |  |  | Lower Bound | Upper Bound |
| 1 | 2 | -,534 | ,227 | ,100 | -1,150 | ,082 |
|  | 3 | 1,387^*^ | ,295 | ,001 | ,584 | 2,190 |
| 2 | 1 | ,534 | ,227 | ,100 | -,082 | 1,150 |
|  | 3 | 1,921^*^ | ,387 | ,001 | ,870 | 2,973 |
| 3 | 1 | -1,387^*^ | ,295 | ,001 | -2,190 | -,584 |
|  | 2 | -1,921^*^ | ,387 | ,001 | -2,973 | -,870 |
| Based on estimated marginal means | | | | | | |
| *. The mean difference is significant at the ,05 level. | | | | | | |
| 1. Adjustment for multiple comparisons: Bonferroni. | | | | | | |
