# Supplementary material for: The effect of changing foot progression angle using real-time visual feedback on rearfoot eversion during running
Source: PLoS One. 2021 Feb 10;16(2):e0246425. doi: 10.1371/journal.pone.0246425 (PMC7875396; doi:10.1371/journal.pone.0246425)
Supplement: S3 Fig — (DOCX) [file pone.0246425.s003.docx]

**S3 Fig**. One-way repeated measure ANOVA results for pronation variables

**A: Peak pronation**

| **Within-Subjects Factors** | |
| --- | --- |
| Measure: MEASURE_1 | |
| FPA | Dependent Variable |
| 1 | Probase_peak |
| 2 | Proplus_peak |
| 3 | Prominus_peak |

| **Descriptive Statistics** | | | |
| --- | --- | --- | --- |
|  | Mean | Std. Deviation | N |
| Probase_peak | 4.4189 | 4.51921 | 15 |
| Proplus_peak | 5.7982 | 4.54803 | 15 |
| Prominus_peak | 2.4216 | 4.63906 | 15 |

| **Tests of Within-Subjects Effects** | | | | | | | |
| --- | --- | --- | --- | --- | --- | --- | --- |
| Measure: MEASURE_1 | | | | | | | |
| Source | | Type III Sum of Squares | df | Mean Square | F | Sig. | Partial Eta Squared |
| FPA | Sphericity Assumed | 86.467 | 2 | 43.234 | 66.356 | .000 | .826 |
|  | Greenhouse-Geisser | 86.467 | 1.314 | 65.821 | 66.356 | .000 | .826 |
|  | Huynh-Feldt | 86.467 | 1.396 | 61.957 | 66.356 | .000 | .826 |
|  | Lower-bound | 86.467 | 1.000 | 86.467 | 66.356 | .000 | .826 |
| Error(FPA) | Sphericity Assumed | 18.243 | 28 | .652 |  |  |  |
|  | Greenhouse-Geisser | 18.243 | 18.391 | .992 |  |  |  |
|  | Huynh-Feldt | 18.243 | 19.539 | .934 |  |  |  |
|  | Lower-bound | 18.243 | 14.000 | 1.303 |  |  |  |

| **Pairwise Comparisons** | | | | | | |
| --- | --- | --- | --- | --- | --- | --- |
| Measure: MEASURE_1 | | | | | | |
| (I) FPA | (J) FPA | Mean Difference (I-J) | Std. Error | Sig.^b^ | 95% Confidence Interval for Difference^b^ | |
|  |  |  |  |  | Lower Bound | Upper Bound |
| 1 | 2 | -1.379^*^ | .314 | .002 | -2.232 | -.527 |
|  | 3 | 1.997^*^ | .166 | .000 | 1.545 | 2.449 |
| 2 | 1 | 1.379^*^ | .314 | .002 | .527 | 2.232 |
|  | 3 | 3.377^*^ | .367 | .000 | 2.380 | 4.374 |
| 3 | 1 | -1.997^*^ | .166 | .000 | -2.449 | -1.545 |
|  | 2 | -3.377^*^ | .367 | .000 | -4.374 | -2.380 |
| Based on estimated marginal means | | | | | | |
| *. The mean difference is significant at the .05 level. | | | | | | |
| b. Adjustment for multiple comparisons: Bonferroni. | | | | | | |

**B: Time to peak pronation**

| **Within-Subjects Factors** | |
| --- | --- |
| Measure: MEASURE_1 | |
| FPA | Dependent Variable |
| 1 | Probase_time |
| 2 | Proplus_time |
| 3 | Prominus_time |

| **Descriptive Statistics** | | | |
| --- | --- | --- | --- |
|  | Mean | Std. Deviation | N |
| Probase_time | 70.07 | 15.215 | 15 |
| Proplus_time | 72.13 | 17.299 | 15 |
| Prominus_time | 69.60 | 15.977 | 15 |

| **Tests of Within-Subjects Effects** | | | | | | | |
| --- | --- | --- | --- | --- | --- | --- | --- |
| Measure: MEASURE_1 | | | | | | | |
| Source | | Type III Sum of Squares | df | Mean Square | F | Sig. | Partial Eta Squared |
| FPA | Sphericity Assumed | 54.533 | 2 | 27.267 | 4.559 | .019 | .246 |
|  | Greenhouse-Geisser | 54.533 | 1.640 | 33.249 | 4.559 | .027 | .246 |
|  | Huynh-Feldt | 54.533 | 1.829 | 29.821 | 4.559 | .023 | .246 |
|  | Lower-bound | 54.533 | 1.000 | 54.533 | 4.559 | .051 | .246 |
| Error(FPA) | Sphericity Assumed | 167.467 | 28 | 5.981 |  |  |  |
|  | Greenhouse-Geisser | 167.467 | 22.962 | 7.293 |  |  |  |
|  | Huynh-Feldt | 167.467 | 25.602 | 6.541 |  |  |  |
|  | Lower-bound | 167.467 | 14.000 | 11.962 |  |  |  |

| **Pairwise Comparisons** | | | | | | |
| --- | --- | --- | --- | --- | --- | --- |
| Measure: MEASURE_1 | | | | | | |
| (I) FPA | (J) FPA | Mean Difference (I-J) | Std. Error | Sig.^b^ | 95% Confidence Interval for Difference^b^ | |
|  |  |  |  |  | Lower Bound | Upper Bound |
| 1 | 2 | -2.067 | 1.067 | .219 | -4.966 | .832 |
|  | 3 | .467 | .703 | 1.000 | -1.443 | 2.377 |
| 2 | 1 | 2.067 | 1.067 | .219 | -.832 | 4.966 |
|  | 3 | 2.533^*^ | .872 | .035 | .163 | 4.904 |
| 3 | 1 | -.467 | .703 | 1.000 | -2.377 | 1.443 |
|  | 2 | -2.533^*^ | .872 | .035 | -4.904 | -.163 |
| Based on estimated marginal means | | | | | | |
| *. The mean difference is significant at the .05 level. | | | | | | |
| b. Adjustment for multiple comparisons: Bonferroni. | | | | | | |

**C: Pronation at touchdown**

| **Within-Subjects Factors** | |
| --- | --- |
| Measure: MEASURE_1 | |
| FPA | Dependent Variable |
| 1 | Probase_TD |
| 2 | Proplus_TD |
| 3 | Prominus_TD |

| **Descriptive Statistics** | | | |
| --- | --- | --- | --- |
|  | Mean | Std. Deviation | N |
| Probase_TD | -2.3335 | 4.86148 | 15 |
| Proplus_TD | -.9653 | 4.78290 | 15 |
| Prominus_TD | -3.5270 | 4.94567 | 15 |

| **Tests of Within-Subjects Effects** | | | | | | | |
| --- | --- | --- | --- | --- | --- | --- | --- |
| Measure: MEASURE_1 | | | | | | | |
| Source | | Type III Sum of Squares | df | Mean Square | F | Sig. | Partial Eta Squared |
| FPA | Sphericity Assumed | 49.291 | 2 | 24.646 | 24.687 | .000 | .638 |
|  | Greenhouse-Geisser | 49.291 | 1.793 | 27.493 | 24.687 | .000 | .638 |
|  | Huynh-Feldt | 49.291 | 2.000 | 24.646 | 24.687 | .000 | .638 |
|  | Lower-bound | 49.291 | 1.000 | 49.291 | 24.687 | .000 | .638 |
| Error(FPA) | Sphericity Assumed | 27.953 | 28 | .998 |  |  |  |
|  | Greenhouse-Geisser | 27.953 | 25.100 | 1.114 |  |  |  |
|  | Huynh-Feldt | 27.953 | 28.000 | .998 |  |  |  |
|  | Lower-bound | 27.953 | 14.000 | 1.997 |  |  |  |

| **Pairwise Comparisons** | | | | | | |
| --- | --- | --- | --- | --- | --- | --- |
| Measure: MEASURE_1 | | | | | | |
| (I) FPA | (J) FPA | Mean Difference (I-J) | Std. Error | Sig.^b^ | 95% Confidence Interval for Difference^b^ | |
|  |  |  |  |  | Lower Bound | Upper Bound |
| 1 | 2 | -1.368^*^ | .412 | .015 | -2.488 | -.248 |
|  | 3 | 1.194^*^ | .303 | .004 | .370 | 2.017 |
| 2 | 1 | 1.368^*^ | .412 | .015 | .248 | 2.488 |
|  | 3 | 2.562^*^ | .371 | .000 | 1.553 | 3.570 |
| 3 | 1 | -1.194^*^ | .303 | .004 | -2.017 | -.370 |
|  | 2 | -2.562^*^ | .371 | .000 | -3.570 | -1.553 |
| Based on estimated marginal means | | | | | | |
| *. The mean difference is significant at the .05 level. | | | | | | |
| b. Adjustment for multiple comparisons: Bonferroni. | | | | | | |

**D: Pronation excursion**

| **Within-Subjects Factors** | |
| --- | --- |
| Measure: MEASURE_1 | |
| FPA | Dependent Variable |
| 1 | Probase_excurs |
| 2 | Proplus_excurs |
| 3 | Prominus_excurs |

| **Descriptive Statistics** | | | |
| --- | --- | --- | --- |
|  | Mean | Std. Deviation | N |
| Probase_excurs | 6.7524 | 4.19076 | 15 |
| Proplus_excurs | 6.7635 | 3.40961 | 15 |
| Prominus_excurs | 5.9485 | 4.01125 | 15 |

| **Tests of Within-Subjects Effects** | | | | | | | |
| --- | --- | --- | --- | --- | --- | --- | --- |
| Measure: MEASURE_1 | | | | | | | |
| Source | | Type III Sum of Squares | df | Mean Square | F | Sig. | Partial Eta Squared |
| FPA | Sphericity Assumed | 6.553 | 2 | 3.276 | 3.566 | .042 | .203 |
|  | Greenhouse-Geisser | 6.553 | 1.848 | 3.545 | 3.566 | .046 | .203 |
|  | Huynh-Feldt | 6.553 | 2.000 | 3.276 | 3.566 | .042 | .203 |
|  | Lower-bound | 6.553 | 1.000 | 6.553 | 3.566 | .080 | .203 |
| Error(FPA) | Sphericity Assumed | 25.722 | 28 | .919 |  |  |  |
|  | Greenhouse-Geisser | 25.722 | 25.877 | .994 |  |  |  |
|  | Huynh-Feldt | 25.722 | 28.000 | .919 |  |  |  |
|  | Lower-bound | 25.722 | 14.000 | 1.837 |  |  |  |

| **Pairwise Comparisons** | | | | | | |
| --- | --- | --- | --- | --- | --- | --- |
| Measure: MEASURE_1 | | | | | | |
| (I) FPA | (J) FPA | Mean Difference (I-J) | Std. Error | Sig.^a^ | 95% Confidence Interval for Difference^a^ | |
|  |  |  |  |  | Lower Bound | Upper Bound |
| 1 | 2 | -.011 | .396 | 1.000 | -1.088 | 1.066 |
|  | 3 | .804 | .316 | .070 | -.055 | 1.663 |
| 2 | 1 | .011 | .396 | 1.000 | -1.066 | 1.088 |
|  | 3 | .815 | .332 | .084 | -.089 | 1.719 |
| 3 | 1 | -.804 | .316 | .070 | -1.663 | .055 |
|  | 2 | -.815 | .332 | .084 | -1.719 | .089 |
| Based on estimated marginal means | | | | | | |
| a. Adjustment for multiple comparisons: Bonferroni. | | | | | | |
