# Supplementary material for: The effect of changing foot progression angle using real-time visual feedback on rearfoot eversion during running
Source: PLoS One. 2021 Feb 10;16(2):e0246425. doi: 10.1371/journal.pone.0246425 (PMC7875396; doi:10.1371/journal.pone.0246425)
Supplement: S4 Fig — (DOCX) [file pone.0246425.s004.docx]

**S4 Fig**. One-way repeated measure ANOVA results for medial longitudinal arch angle (MLAA)

A: peak MLAA

| **Within-Subjects Factors** | |
| --- | --- |
| Measure: MEASURE_1 | |
| FPA | Dependent Variable |
| 1 | MLAAbase_peak |
| 2 | MLAAplus_peak |
| 3 | MLAAminus_peak |

| **Descriptive Statistics** | | | |
| --- | --- | --- | --- |
|  | Mean | Std. Deviation | N |
| MLAAbase_peak | 6,1639 | 2,15790 | 15 |
| MLAAplus_peak | 6,3680 | 2,24932 | 15 |
| MLAAminus_peak | 5,4673 | 2,32266 | 15 |

| **Tests of Within-Subjects Effects** | | | | | | | |
| --- | --- | --- | --- | --- | --- | --- | --- |
| Measure: MEASURE_1 | | | | | | | |
| Source | | Type III Sum of Squares | df | Mean Square | F | Sig. | Partial Eta Squared |
| FPA | Sphericity Assumed | 6,692 | 2 | 3,346 | 9,699 | ,001 | ,409 |
|  | Greenhouse-Geisser | 6,692 | 1,881 | 3,557 | 9,699 | ,001 | ,409 |
|  | Huynh-Feldt | 6,692 | 2,000 | 3,346 | 9,699 | ,001 | ,409 |
|  | Lower-bound | 6,692 | 1,000 | 6,692 | 9,699 | ,008 | ,409 |
| Error(FPA) | Sphericity Assumed | 9,659 | 28 | ,345 |  |  |  |
|  | Greenhouse-Geisser | 9,659 | 26,334 | ,367 |  |  |  |
|  | Huynh-Feldt | 9,659 | 28,000 | ,345 |  |  |  |
|  | Lower-bound | 9,659 | 14,000 | ,690 |  |  |  |

| **Pairwise Comparisons** | | | | | | |
| --- | --- | --- | --- | --- | --- | --- |
| Measure: MEASURE_1 | | | | | | |
| (I) FPA | (J) FPA | Mean Difference (I-J) | Std. Error | Sig.^b^ | 95% Confidence Interval for Difference^b^ | |
|  |  |  |  |  | Lower Bound | Upper Bound |
| 1 | 2 | -,204 | ,186 | ,876 | -,711 | ,302 |
|  | 3 | ,697^*^ | ,222 | ,022 | ,093 | 1,300 |
| 2 | 1 | ,204 | ,186 | ,876 | -,302 | ,711 |
|  | 3 | ,901^*^ | ,232 | ,005 | ,269 | 1,532 |
| 3 | 1 | -,697^*^ | ,222 | ,022 | -1,300 | -,093 |
|  | 2 | -,901^*^ | ,232 | ,005 | -1,532 | -,269 |
| Based on estimated marginal means | | | | | | |
| *. The mean difference is significant at the ,05 level. | | | | | | |
| b. Adjustment for multiple comparisons: Bonferroni. | | | | | | |

B: Time to peak MLAA

| **Within-Subjects Factors** | |
| --- | --- |
| Measure: MEASURE_1 | |
| FPA | Dependent Variable |
| 1 | MLAAbase_time |
| 2 | MLAAplus_time |
| 3 | MLAAminus_time |

| **Descriptive Statistics** | | | |
| --- | --- | --- | --- |
|  | Mean | Std. Deviation | N |
| MLAAbase_time | 54,00 | 8,071 | 15 |
| MLAAplus_time | 56,60 | 7,099 | 15 |
| MLAAminus_time | 54,13 | 9,156 | 15 |

| **Tests of Within-Subjects Effects** | | | | | | | |
| --- | --- | --- | --- | --- | --- | --- | --- |
| Measure: MEASURE_1 | | | | | | | |
| Source | | Type III Sum of Squares | df | Mean Square | F | Sig. | Partial Eta Squared |
| FPA | Sphericity Assumed | 64,311 | 2 | 32,156 | 3,606 | ,040 | ,205 |
|  | Greenhouse-Geisser | 64,311 | 1,680 | 38,278 | 3,606 | ,050 | ,205 |
|  | Huynh-Feldt | 64,311 | 1,883 | 34,149 | 3,606 | ,044 | ,205 |
|  | Lower-bound | 64,311 | 1,000 | 64,311 | 3,606 | ,078 | ,205 |
| Error(FPA) | Sphericity Assumed | 249,689 | 28 | 8,917 |  |  |  |
|  | Greenhouse-Geisser | 249,689 | 23,521 | 10,615 |  |  |  |
|  | Huynh-Feldt | 249,689 | 26,365 | 9,470 |  |  |  |
|  | Lower-bound | 249,689 | 14,000 | 17,835 |  |  |  |

| **Pairwise Comparisons** | | | | | | |
| --- | --- | --- | --- | --- | --- | --- |
| Measure: MEASURE_1 | | | | | | |
| (I) FPA | (J) FPA | Mean Difference (I-J) | Std. Error | Sig.^b^ | 95% Confidence Interval for Difference^b^ | |
|  |  |  |  |  | Lower Bound | Upper Bound |
| 1 | 2 | -2,600^*^ | ,888 | ,033 | -5,013 | -,187 |
|  | 3 | -,133 | 1,294 | 1,000 | -3,651 | 3,384 |
| 2 | 1 | 2,600^*^ | ,888 | ,033 | ,187 | 5,013 |
|  | 3 | 2,467 | 1,050 | ,102 | -,388 | 5,322 |
| 3 | 1 | ,133 | 1,294 | 1,000 | -3,384 | 3,651 |
|  | 2 | -2,467 | 1,050 | ,102 | -5,322 | ,388 |
| Based on estimated marginal means | | | | | | |
| *. The mean difference is significant at the ,05 level. | | | | | | |
| b. Adjustment for multiple comparisons: Bonferroni. | | | | | | |

**C: MLAA at touchdown**

| **Within-Subjects Factors** | |
| --- | --- |
| Measure: MEASURE_1 | |
| FPA | Dependent Variable |
| 1 | MLAAbase_TD |
| 2 | MLAAplus_TD |
| 3 | MLAAminus_TD |

| **Descriptive Statistics** | | | |
| --- | --- | --- | --- |
|  | Mean | Std. Deviation | N |
| MLAAbase_TD | -1,006 | 2,2969 | 15 |
| MLAAplus_TD | -1,028 | 2,5336 | 15 |
| MLAAminus_TD | -,923 | 2,7182 | 15 |

| **Tests of Within-Subjects Effects** | | | | | | | |
| --- | --- | --- | --- | --- | --- | --- | --- |
| Measure: MEASURE_1 | | | | | | | |
| Source | | Type III Sum of Squares | df | Mean Square | F | Sig. | Partial Eta Squared |
| FPA | Sphericity Assumed | ,093 | 2 | ,047 | ,205 | ,816 | ,014 |
|  | Greenhouse-Geisser | ,093 | 1,334 | ,070 | ,205 | ,726 | ,014 |
|  | Huynh-Feldt | ,093 | 1,422 | ,066 | ,205 | ,741 | ,014 |
|  | Lower-bound | ,093 | 1,000 | ,093 | ,205 | ,658 | ,014 |
| Error(FPA) | Sphericity Assumed | 6,392 | 28 | ,228 |  |  |  |
|  | Greenhouse-Geisser | 6,392 | 18,675 | ,342 |  |  |  |
|  | Huynh-Feldt | 6,392 | 19,905 | ,321 |  |  |  |
|  | Lower-bound | 6,392 | 14,000 | ,457 |  |  |  |

| **Pairwise Comparisons** | | | | | | |
| --- | --- | --- | --- | --- | --- | --- |
| Measure: MEASURE_1 | | | | | | |
| (I) FPA | (J) FPA | Mean Difference (I-J) | Std. Error | Sig.^a^ | 95% Confidence Interval for Difference^a^ | |
|  |  |  |  |  | Lower Bound | Upper Bound |
| 1 | 2 | ,022 | ,165 | 1,000 | -,425 | ,469 |
|  | 3 | -,084 | ,225 | 1,000 | -,695 | ,527 |
| 2 | 1 | -,022 | ,165 | 1,000 | -,469 | ,425 |
|  | 3 | -,106 | ,117 | 1,000 | -,424 | ,213 |
| 3 | 1 | ,084 | ,225 | 1,000 | -,527 | ,695 |
|  | 2 | ,106 | ,117 | 1,000 | -,213 | ,424 |
| Based on estimated marginal means | | | | | | |
| a. Adjustment for multiple comparisons: Bonferroni. | | | | | | |

**D: MLAA excursion**

| **Within-Subjects Factors** | |
| --- | --- |
| Measure: MEASURE_1 | |
| FPA | Dependent Variable |
| 1 | MLAAbase_excurs |
| 2 | MLAAplus_excurs |
| 3 | MLAAminus_excurs |

| **Descriptive Statistics** | | | |
| --- | --- | --- | --- |
|  | Mean | Std. Deviation | N |
| MLAAbase_excurs | 7,170 | 1,6685 | 15 |
| MLAAplus_excurs | 7,396 | 1,6655 | 15 |
| MLAAminus_excurs | 6,390 | 1,7021 | 15 |

| **Tests of Within-Subjects Effects** | | | | | | | |
| --- | --- | --- | --- | --- | --- | --- | --- |
| Measure: MEASURE_1 | | | | | | | |
| Source | | Type III Sum of Squares | df | Mean Square | F | Sig. | Partial Eta Squared |
| FPA | Sphericity Assumed | 8,366 | 2 | 4,183 | 13,018 | ,000 | ,482 |
|  | Greenhouse-Geisser | 8,366 | 1,684 | 4,967 | 13,018 | ,000 | ,482 |
|  | Huynh-Feldt | 8,366 | 1,889 | 4,429 | 13,018 | ,000 | ,482 |
|  | Lower-bound | 8,366 | 1,000 | 8,366 | 13,018 | ,003 | ,482 |
| Error(FPA) | Sphericity Assumed | 8,997 | 28 | ,321 |  |  |  |
|  | Greenhouse-Geisser | 8,997 | 23,579 | ,382 |  |  |  |
|  | Huynh-Feldt | 8,997 | 26,445 | ,340 |  |  |  |
|  | Lower-bound | 8,997 | 14,000 | ,643 |  |  |  |

| **Pairwise Comparisons** | | | | | | |
| --- | --- | --- | --- | --- | --- | --- |
| Measure: MEASURE_1 | | | | | | |
| (I) FPA | (J) FPA | Mean Difference (I-J) | Std. Error | Sig.^b^ | 95% Confidence Interval for Difference^b^ | |
|  |  |  |  |  | Lower Bound | Upper Bound |
| 1 | 2 | -,226 | ,244 | 1,000 | -,890 | ,438 |
|  | 3 | ,780^*^ | ,203 | ,005 | ,229 | 1,331 |
| 2 | 1 | ,226 | ,244 | 1,000 | -,438 | ,890 |
|  | 3 | 1,007^*^ | ,166 | ,000 | ,554 | 1,459 |
| 3 | 1 | -,780^*^ | ,203 | ,005 | -1,331 | -,229 |
|  | 2 | -1,007^*^ | ,166 | ,000 | -1,459 | -,554 |
| Based on estimated marginal means | | | | | | |
| *. The mean difference is significant at the ,05 level. | | | | | | |
| b. Adjustment for multiple comparisons: Bonferroni. | | | | | | |
