# Supplementary material for: The effect of changing foot progression angle using real-time visual feedback on rearfoot eversion during running
Source: PLoS One. 2021 Feb 10;16(2):e0246425. doi: 10.1371/journal.pone.0246425 (PMC7875396; doi:10.1371/journal.pone.0246425)
Supplement: S5 Fig — (DOCX) [file pone.0246425.s005.docx]

**S5 Fig**. One-way repeated measure ANOVA results for hip internal/external rotation

1. **Peak hip internal rotation**

| **Within-Subjects Factors** | |
| --- | --- |
| Measure: MEASURE_1 | |
| FPA | Dependent Variable |
| 1 | HrotBase_peak |
| 2 | HrotPlus_peak |
| 3 | HrotMinus_peak |

| **Descriptive Statistics** | | | |
| --- | --- | --- | --- |
|  | Mean | Std. Deviation | N |
| HrotBase_peak | 7.5819 | 4.77828 | 15 |
| HrotPlus_peak | 5.0711 | 5.21468 | 15 |
| HrotMinus_peak | 9.8387 | 5.07714 | 15 |

| **Tests of Within-Subjects Effects** | | | | | | | |
| --- | --- | --- | --- | --- | --- | --- | --- |
| Measure: MEASURE_1 | | | | | | | |
| Source | | Type III Sum of Squares | df | Mean Square | F | Sig. | Partial Eta Squared |
| FPA | Sphericity Assumed | 170.635 | 2 | 85.317 | 71.275 | .000 | .836 |
|  | Greenhouse-Geisser | 170.635 | 1.681 | 101.513 | 71.275 | .000 | .836 |
|  | Huynh-Feldt | 170.635 | 1.884 | 90.552 | 71.275 | .000 | .836 |
|  | Lower-bound | 170.635 | 1.000 | 170.635 | 71.275 | .000 | .836 |
| Error(FPA) | Sphericity Assumed | 33.517 | 28 | 1.197 |  |  |  |
|  | Greenhouse-Geisser | 33.517 | 23.533 | 1.424 |  |  |  |
|  | Huynh-Feldt | 33.517 | 26.381 | 1.270 |  |  |  |
|  | Lower-bound | 33.517 | 14.000 | 2.394 |  |  |  |

| **Pairwise Comparisons** | | | | | | |
| --- | --- | --- | --- | --- | --- | --- |
| Measure: MEASURE_1 | | | | | | |
| (I) FPA | (J) FPA | Mean Difference (I-J) | Std. Error | Sig.^b^ | 95% Confidence Interval for Difference^b^ | |
|  |  |  |  |  | Lower Bound | Upper Bound |
| 1 | 2 | 2.511^*^ | .311 | .000 | 1.664 | 3.357 |
|  | 3 | -2.257^*^ | .406 | .000 | -3.360 | -1.154 |
| 2 | 1 | -2.511^*^ | .311 | .000 | -3.357 | -1.664 |
|  | 3 | -4.768^*^ | .466 | .000 | -6.034 | -3.501 |
| 3 | 1 | 2.257^*^ | .406 | .000 | 1.154 | 3.360 |
|  | 2 | 4.768^*^ | .466 | .000 | 3.501 | 6.034 |
| Based on estimated marginal means | | | | | | |
| *. The mean difference is significant at the .05 level. | | | | | | |
| b. Adjustment for multiple comparisons: Bonferroni. | | | | | | |

**B. Time to peak hip internal rotation**

| **Within-Subjects Factors** | |
| --- | --- |
| Measure: MEASURE_1 | |
| FPA | Dependent Variable |
| 1 | HrotBase_time |
| 2 | HrotPlus_time |
| 3 | HrotMinus_time |

| **Descriptive Statistics** | | | |
| --- | --- | --- | --- |
|  | Mean | Std. Deviation | N |
| HrotBase_time | 58.33 | 38.818 | 15 |
| HrotPlus_time | 59.87 | 35.737 | 15 |
| HrotMinus_time | 51.93 | 39.593 | 15 |

| **Tests of Within-Subjects Effects** | | | | | | | |
| --- | --- | --- | --- | --- | --- | --- | --- |
| Measure: MEASURE_1 | | | | | | | |
| Source | | Type III Sum of Squares | df | Mean Square | F | Sig. | Partial Eta Squared |
| FPA | Sphericity Assumed | 531.244 | 2 | 265.622 | 1.695 | .202 | .108 |
|  | Greenhouse-Geisser | 531.244 | 1.139 | 466.257 | 1.695 | .214 | .108 |
|  | Huynh-Feldt | 531.244 | 1.173 | 452.737 | 1.695 | .213 | .108 |
|  | Lower-bound | 531.244 | 1.000 | 531.244 | 1.695 | .214 | .108 |
| Error(FPA) | Sphericity Assumed | 4386.756 | 28 | 156.670 |  |  |  |
|  | Greenhouse-Geisser | 4386.756 | 15.951 | 275.009 |  |  |  |
|  | Huynh-Feldt | 4386.756 | 16.428 | 267.034 |  |  |  |
|  | Lower-bound | 4386.756 | 14.000 | 313.340 |  |  |  |

| **Pairwise Comparisons** | | | | | | |
| --- | --- | --- | --- | --- | --- | --- |
| Measure: MEASURE_1 | | | | | | |
| (I) FPA | (J) FPA | Mean Difference (I-J) | Std. Error | Sig.^a^ | 95% Confidence Interval for Difference^a^ | |
|  |  |  |  |  | Lower Bound | Upper Bound |
| 1 | 2 | -1.533 | 1.924 | 1.000 | -6.764 | 3.697 |
|  | 3 | 6.400 | 5.878 | .884 | -9.575 | 22.375 |
| 2 | 1 | 1.533 | 1.924 | 1.000 | -3.697 | 6.764 |
|  | 3 | 7.933 | 4.941 | .392 | -5.495 | 21.362 |
| 3 | 1 | -6.400 | 5.878 | .884 | -22.375 | 9.575 |
|  | 2 | -7.933 | 4.941 | .392 | -21.362 | 5.495 |
| Based on estimated marginal means | | | | | | |
| a. Adjustment for multiple comparisons: Bonferroni. | | | | | | |

**C. Hip internal rotation at touchdown**

| **Within-Subjects Factors** | |
| --- | --- |
| Measure: MEASURE_1 | |
| FPA | Dependent Variable |
| 1 | hrotBase_TD |
| 2 | HrotPlus_TD |
| 3 | HrotMinus_TD |

| **Descriptive Statistics** | | | |
| --- | --- | --- | --- |
|  | Mean | Std. Deviation | N |
| hrotBase_TD | 4.5437 | 5.70777 | 15 |
| HrotPlus_TD | 1.5747 | 5.94454 | 15 |
| HrotMinus_TD | 7.0066 | 5.78944 | 15 |

| **Tests of Within-Subjects Effects** | | | | | | | |
| --- | --- | --- | --- | --- | --- | --- | --- |
| Measure: MEASURE_1 | | | | | | | |
| Source | | Type III Sum of Squares | df | Mean Square | F | Sig. | Partial Eta Squared |
| FPA | Sphericity Assumed | 221.928 | 2 | 110.964 | 82.251 | .000 | .855 |
|  | Greenhouse-Geisser | 221.928 | 1.767 | 125.589 | 82.251 | .000 | .855 |
|  | Huynh-Feldt | 221.928 | 2.000 | 110.964 | 82.251 | .000 | .855 |
|  | Lower-bound | 221.928 | 1.000 | 221.928 | 82.251 | .000 | .855 |
| Error(FPA) | Sphericity Assumed | 37.775 | 28 | 1.349 |  |  |  |
|  | Greenhouse-Geisser | 37.775 | 24.739 | 1.527 |  |  |  |
|  | Huynh-Feldt | 37.775 | 28.000 | 1.349 |  |  |  |
|  | Lower-bound | 37.775 | 14.000 | 2.698 |  |  |  |

| **Pairwise Comparisons** | | | | | | |
| --- | --- | --- | --- | --- | --- | --- |
| Measure: MEASURE_1 | | | | | | |
| (I) FPA | (J) FPA | Mean Difference (I-J) | Std. Error | Sig.^b^ | 95% Confidence Interval for Difference^b^ | |
|  |  |  |  |  | Lower Bound | Upper Bound |
| 1 | 2 | 2.969^*^ | .351 | .000 | 2.016 | 3.922 |
|  | 3 | -2.463^*^ | .425 | .000 | -3.618 | -1.308 |
| 2 | 1 | -2.969^*^ | .351 | .000 | -3.922 | -2.016 |
|  | 3 | -5.432^*^ | .486 | .000 | -6.752 | -4.111 |
| 3 | 1 | 2.463^*^ | .425 | .000 | 1.308 | 3.618 |
|  | 2 | 5.432^*^ | .486 | .000 | 4.111 | 6.752 |
| Based on estimated marginal means | | | | | | |
| *. The mean difference is significant at the .05 level. | | | | | | |
| b. Adjustment for multiple comparisons: Bonferroni. | | | | | | |

**D. Hip internal rotation excursion**

| **Within-Subjects Factors** | |
| --- | --- |
| Measure: MEASURE_1 | |
| FPA | Dependent Variable |
| 1 | HrotBase_excur |
| 2 | HrotPlus_excur |
| 3 | HrotMinus_excur |

| **Descriptive Statistics** | | | |
| --- | --- | --- | --- |
|  | Mean | Std. Deviation | N |
| HrotBase_excur | 3.0382 | 2.71573 | 15 |
| HrotPlus_excur | 3.4964 | 2.45773 | 15 |
| HrotMinus_excur | 2.8321 | 2.98208 | 15 |

| **Tests of Within-Subjects Effects** | | | | | | | |
| --- | --- | --- | --- | --- | --- | --- | --- |
| Measure: MEASURE_1 | | | | | | | |
| Source | | Type III Sum of Squares | df | Mean Square | F | Sig. | Partial Eta Squared |
| FPA | Sphericity Assumed | 3.468 | 2 | 1.734 | 1.212 | .313 | .080 |
|  | Greenhouse-Geisser | 3.468 | 1.460 | 2.376 | 1.212 | .305 | .080 |
|  | Huynh-Feldt | 3.468 | 1.586 | 2.186 | 1.212 | .307 | .080 |
|  | Lower-bound | 3.468 | 1.000 | 3.468 | 1.212 | .290 | .080 |
| Error(FPA) | Sphericity Assumed | 40.072 | 28 | 1.431 |  |  |  |
|  | Greenhouse-Geisser | 40.072 | 20.433 | 1.961 |  |  |  |
|  | Huynh-Feldt | 40.072 | 22.208 | 1.804 |  |  |  |
|  | Lower-bound | 40.072 | 14.000 | 2.862 |  |  |  |

| **Pairwise Comparisons** | | | | | | |
| --- | --- | --- | --- | --- | --- | --- |
| Measure: MEASURE_1 | | | | | | |
| (I) FPA | (J) FPA | Mean Difference (I-J) | Std. Error | Sig.^a^ | 95% Confidence Interval for Difference^a^ | |
|  |  |  |  |  | Lower Bound | Upper Bound |
| 1 | 2 | -.458 | .332 | .569 | -1.361 | .445 |
|  | 3 | .206 | .551 | 1.000 | -1.291 | 1.704 |
| 2 | 1 | .458 | .332 | .569 | -.445 | 1.361 |
|  | 3 | .664 | .398 | .352 | -.417 | 1.746 |
| 3 | 1 | -.206 | .551 | 1.000 | -1.704 | 1.291 |
|  | 2 | -.664 | .398 | .352 | -1.746 | .417 |
| Based on estimated marginal means | | | | | | |
| a. Adjustment for multiple comparisons: Bonferroni. | | | | | | |
