# Supplementary material for: The effect of changing foot progression angle using real-time visual feedback on rearfoot eversion during running
Source: PLoS One. 2021 Feb 10;16(2):e0246425. doi: 10.1371/journal.pone.0246425 (PMC7875396; doi:10.1371/journal.pone.0246425)
Supplement: S6 Fig — (DOCX) [file pone.0246425.s006.docx]

**S6 Fig**. One-way repeated measure ANOVA results for hip ab/adduction

**A. Peak hip ab/adduction**

| **Within-Subjects Factors** | |
| --- | --- |
| Measure: MEASURE_1 | |
| FPA | Dependent Variable |
| 1 | HabdBase_peak |
| 2 | HabdPlus_peak |
| 3 | HabdMinus_peak |

| **Descriptive Statistics** | | | |
| --- | --- | --- | --- |
|  | Mean | Std. Deviation | N |
| HabdBase_peak | 14.0258 | 3.56366 | 15 |
| HabdPlus_peak | 11.5528 | 3.36939 | 15 |
| HabdMinus_peak | 12.9995 | 4.62887 | 15 |

| **Tests of Within-Subjects Effects** | | | | | | | |
| --- | --- | --- | --- | --- | --- | --- | --- |
| Measure: MEASURE_1 | | | | | | | |
| Source | | Type III Sum of Squares | df | Mean Square | F | Sig. | Partial Eta Squared |
| FPA | Sphericity Assumed | 46.306 | 2 | 23.153 | 12.376 | .000 | .469 |
|  | Greenhouse-Geisser | 46.306 | 1.668 | 27.760 | 12.376 | .000 | .469 |
|  | Huynh-Feldt | 46.306 | 1.867 | 24.805 | 12.376 | .000 | .469 |
|  | Lower-bound | 46.306 | 1.000 | 46.306 | 12.376 | .003 | .469 |
| Error(FPA) | Sphericity Assumed | 52.383 | 28 | 1.871 |  |  |  |
|  | Greenhouse-Geisser | 52.383 | 23.354 | 2.243 |  |  |  |
|  | Huynh-Feldt | 52.383 | 26.136 | 2.004 |  |  |  |
|  | Lower-bound | 52.383 | 14.000 | 3.742 |  |  |  |

| **Pairwise Comparisons** | | | | | | |
| --- | --- | --- | --- | --- | --- | --- |
| Measure: MEASURE_1 | | | | | | |
| (I) FPA | (J) FPA | Mean Difference (I-J) | Std. Error | Sig.^b^ | 95% Confidence Interval for Difference^b^ | |
|  |  |  |  |  | Lower Bound | Upper Bound |
| 1 | 2 | 2.473^*^ | .380 | .000 | 1.440 | 3.506 |
|  | 3 | 1.026 | .578 | .292 | -.544 | 2.597 |
| 2 | 1 | -2.473^*^ | .380 | .000 | -3.506 | -1.440 |
|  | 3 | -1.447^*^ | .519 | .044 | -2.858 | -.035 |
| 3 | 1 | -1.026 | .578 | .292 | -2.597 | .544 |
|  | 2 | 1.447^*^ | .519 | .044 | .035 | 2.858 |
| Based on estimated marginal means | | | | | | |
| *. The mean difference is significant at the .05 level. | | | | | | |
| b. Adjustment for multiple comparisons: Bonferroni. | | | | | | |

**B. Time to peak hip ab/adduction**

| **Within-Subjects Factors** | |
| --- | --- |
| Measure: MEASURE_1 | |
| FPA | Dependent Variable |
| 1 | HabdBase_time |
| 2 | HabdPlus_time |
| 3 | HabdMinus_time |

| **Descriptive Statistics** | | | |
| --- | --- | --- | --- |
|  | Mean | Std. Deviation | N |
| HabdBase_time | 43.20 | 3.570 | 15 |
| HabdPlus_time | 43.40 | 4.032 | 15 |
| HabdMinus_time | 45.73 | 5.483 | 15 |

| **Tests of Within-Subjects Effects** | | | | | | | |
| --- | --- | --- | --- | --- | --- | --- | --- |
| Measure: MEASURE_1 | | | | | | | |
| Source | | Type III Sum of Squares | df | Mean Square | F | Sig. | Partial Eta Squared |
| FPA | Sphericity Assumed | 59.511 | 2 | 29.756 | 4.022 | .029 | .223 |
|  | Greenhouse-Geisser | 59.511 | 1.209 | 49.243 | 4.022 | .055 | .223 |
|  | Huynh-Feldt | 59.511 | 1.261 | 47.200 | 4.022 | .053 | .223 |
|  | Lower-bound | 59.511 | 1.000 | 59.511 | 4.022 | .065 | .223 |
| Error(FPA) | Sphericity Assumed | 207.156 | 28 | 7.398 |  |  |  |
|  | Greenhouse-Geisser | 207.156 | 16.919 | 12.244 |  |  |  |
|  | Huynh-Feldt | 207.156 | 17.652 | 11.736 |  |  |  |
|  | Lower-bound | 207.156 | 14.000 | 14.797 |  |  |  |

| **Pairwise Comparisons** | | | | | | |
| --- | --- | --- | --- | --- | --- | --- |
| Measure: MEASURE_1 | | | | | | |
| (I) FPA | (J) FPA | Mean Difference (I-J) | Std. Error | Sig.^a^ | 95% Confidence Interval for Difference^a^ | |
|  |  |  |  |  | Lower Bound | Upper Bound |
| 1 | 2 | -.200 | .460 | 1.000 | -1.450 | 1.050 |
|  | 3 | -2.533 | 1.099 | .111 | -5.521 | .454 |
| 2 | 1 | .200 | .460 | 1.000 | -1.050 | 1.450 |
|  | 3 | -2.333 | 1.241 | .243 | -5.706 | 1.039 |
| 3 | 1 | 2.533 | 1.099 | .111 | -.454 | 5.521 |
|  | 2 | 2.333 | 1.241 | .243 | -1.039 | 5.706 |
| Based on estimated marginal means | | | | | | |
| a. Adjustment for multiple comparisons: Bonferroni. | | | | | | |

**C. Hip ab/adduction at touchdown**

| **Within-Subjects Factors** | |
| --- | --- |
| Measure: MEASURE_1 | |
| FPA | Dependent Variable |
| 1 | HabdBase_TD |
| 2 | HabdPlus_TD |
| 3 | HabdMinus_TD |

| **Descriptive Statistics** | | | |
| --- | --- | --- | --- |
|  | Mean | Std. Deviation | N |
| HabdBase_TD | 7.1306 | 2.55733 | 15 |
| HabdPlus_TD | 6.2553 | 2.43448 | 15 |
| HabdMinus_TD | 6.0262 | 3.33063 | 15 |

| **Tests of Within-Subjects Effects** | | | | | | | |
| --- | --- | --- | --- | --- | --- | --- | --- |
| Measure: MEASURE_1 | | | | | | | |
| Source | | Type III Sum of Squares | df | Mean Square | F | Sig. | Partial Eta Squared |
| FPA | Sphericity Assumed | 10.192 | 2 | 5.096 | 3.059 | .063 | .179 |
|  | Greenhouse-Geisser | 10.192 | 1.653 | 6.167 | 3.059 | .075 | .179 |
|  | Huynh-Feldt | 10.192 | 1.846 | 5.522 | 3.059 | .068 | .179 |
|  | Lower-bound | 10.192 | 1.000 | 10.192 | 3.059 | .102 | .179 |
| Error(FPA) | Sphericity Assumed | 46.638 | 28 | 1.666 |  |  |  |
|  | Greenhouse-Geisser | 46.638 | 23.139 | 2.016 |  |  |  |
|  | Huynh-Feldt | 46.638 | 25.842 | 1.805 |  |  |  |
|  | Lower-bound | 46.638 | 14.000 | 3.331 |  |  |  |

| **Pairwise Comparisons** | | | | | | |
| --- | --- | --- | --- | --- | --- | --- |
| Measure: MEASURE_1 | | | | | | |
| (I) FPA | (J) FPA | Mean Difference (I-J) | Std. Error | Sig.^a^ | 95% Confidence Interval for Difference^a^ | |
|  |  |  |  |  | Lower Bound | Upper Bound |
| 1 | 2 | .875 | .425 | .176 | -.280 | 2.031 |
|  | 3 | 1.104 | .569 | .217 | -.441 | 2.650 |
| 2 | 1 | -.875 | .425 | .176 | -2.031 | .280 |
|  | 3 | .229 | .403 | 1.000 | -.865 | 1.323 |
| 3 | 1 | -1.104 | .569 | .217 | -2.650 | .441 |
|  | 2 | -.229 | .403 | 1.000 | -1.323 | .865 |
| Based on estimated marginal means | | | | | | |
| a. Adjustment for multiple comparisons: Bonferroni. | | | | | | |

**D. Hip ab/adduction excursion**

| **Within-Subjects Factors** | |
| --- | --- |
| Measure: MEASURE_1 | |
| FPA | Dependent Variable |
| 1 | HabdBase_excur |
| 2 | HabdPlus_excur |
| 3 | HabdMinus_excur |

| **Descriptive Statistics** | | | |
| --- | --- | --- | --- |
|  | Mean | Std. Deviation | N |
| HabdBase_excur | 6.8951 | 2.52275 | 15 |
| HabdPlus_excur | 5.2976 | 2.26411 | 15 |
| HabdMinus_excur | 6.9733 | 2.89160 | 15 |

| **Tests of Within-Subjects Effects** | | | | | | | |
| --- | --- | --- | --- | --- | --- | --- | --- |
| Measure: MEASURE_1 | | | | | | | |
| Source | | Type III Sum of Squares | df | Mean Square | F | Sig. | Partial Eta Squared |
| FPA | Sphericity Assumed | 26.831 | 2 | 13.415 | 28.451 | .000 | .670 |
|  | Greenhouse-Geisser | 26.831 | 1.782 | 15.057 | 28.451 | .000 | .670 |
|  | Huynh-Feldt | 26.831 | 2.000 | 13.415 | 28.451 | .000 | .670 |
|  | Lower-bound | 26.831 | 1.000 | 26.831 | 28.451 | .000 | .670 |
| Error(FPA) | Sphericity Assumed | 13.203 | 28 | .472 |  |  |  |
|  | Greenhouse-Geisser | 13.203 | 24.946 | .529 |  |  |  |
|  | Huynh-Feldt | 13.203 | 28.000 | .472 |  |  |  |
|  | Lower-bound | 13.203 | 14.000 | .943 |  |  |  |

| **Pairwise Comparisons** | | | | | | |
| --- | --- | --- | --- | --- | --- | --- |
| Measure: MEASURE_1 | | | | | | |
| (I) FPA | (J) FPA | Mean Difference (I-J) | Std. Error | Sig.^b^ | 95% Confidence Interval for Difference^b^ | |
|  |  |  |  |  | Lower Bound | Upper Bound |
| 1 | 2 | 1.598^*^ | .205 | .000 | 1.040 | 2.155 |
|  | 3 | -.078 | .282 | 1.000 | -.845 | .689 |
| 2 | 1 | -1.598^*^ | .205 | .000 | -2.155 | -1.040 |
|  | 3 | -1.676^*^ | .259 | .000 | -2.378 | -.973 |
| 3 | 1 | .078 | .282 | 1.000 | -.689 | .845 |
|  | 2 | 1.676^*^ | .259 | .000 | .973 | 2.378 |
| Based on estimated marginal means | | | | | | |
| *. The mean difference is significant at the .05 level. | | | | | | |
| b. Adjustment for multiple comparisons: Bonferroni. | | | | | | |
