# Supplementary material for: The effect of changing foot progression angle using real-time visual feedback on rearfoot eversion during running
Source: PLoS One. 2021 Feb 10;16(2):e0246425. doi: 10.1371/journal.pone.0246425 (PMC7875396; doi:10.1371/journal.pone.0246425)
Supplement: S7 Fig — (DOCX) [file pone.0246425.s007.docx]

**S7 Fig**. One-way repeated measure ANOVA results for hip flexion

**A. Peak hip flexion**

| **Within-Subjects Factors** | |
| --- | --- |
| Measure: MEASURE_1 | |
| FPA | Dependent Variable |
| 1 | HflexBase_peak |
| 2 | HflexPlus_peak |
| 3 | HflexMinus_peak |

| **Descriptive Statistics** | | | |
| --- | --- | --- | --- |
|  | Mean | Std. Deviation | N |
| HflexBase_peak | 36.7853 | 5.46752 | 15 |
| HflexPlus_peak | 37.0629 | 5.64972 | 15 |
| HflexMinus_peak | 36.4900 | 5.19622 | 15 |

| **Tests of Within-Subjects Effects** | | | | | | | |
| --- | --- | --- | --- | --- | --- | --- | --- |
| Measure: MEASURE_1 | | | | | | | |
| Source | | Type III Sum of Squares | df | Mean Square | F | Sig. | Partial Eta Squared |
| FPA | Sphericity Assumed | 2.462 | 2 | 1.231 | .800 | .460 | .054 |
|  | Greenhouse-Geisser | 2.462 | 1.920 | 1.283 | .800 | .455 | .054 |
|  | Huynh-Feldt | 2.462 | 2.000 | 1.231 | .800 | .460 | .054 |
|  | Lower-bound | 2.462 | 1.000 | 2.462 | .800 | .386 | .054 |
| Error(FPA) | Sphericity Assumed | 43.117 | 28 | 1.540 |  |  |  |
|  | Greenhouse-Geisser | 43.117 | 26.879 | 1.604 |  |  |  |
|  | Huynh-Feldt | 43.117 | 28.000 | 1.540 |  |  |  |
|  | Lower-bound | 43.117 | 14.000 | 3.080 |  |  |  |

| **Pairwise Comparisons** | | | | | | |
| --- | --- | --- | --- | --- | --- | --- |
| Measure: MEASURE_1 | | | | | | |
| (I) FPA | (J) FPA | Mean Difference (I-J) | Std. Error | Sig.^a^ | 95% Confidence Interval for Difference^a^ | |
|  |  |  |  |  | Lower Bound | Upper Bound |
| 1 | 2 | -.278 | .418 | 1.000 | -1.414 | .859 |
|  | 3 | .295 | .442 | 1.000 | -.907 | 1.498 |
| 2 | 1 | .278 | .418 | 1.000 | -.859 | 1.414 |
|  | 3 | .573 | .495 | .801 | -.774 | 1.919 |
| 3 | 1 | -.295 | .442 | 1.000 | -1.498 | .907 |
|  | 2 | -.573 | .495 | .801 | -1.919 | .774 |
| Based on estimated marginal means | | | | | | |
| a. Adjustment for multiple comparisons: Bonferroni. | | | | | | |

**B. time to peak hip flexion**

| **Within-Subjects Factors** | |
| --- | --- |
| Measure: MEASURE_1 | |
| FPA | Dependent Variable |
| 1 | HflexBase_time |
| 2 | HflexPlus_time |
| 3 | HflexMinus_time |

| **Descriptive Statistics** | | | |
| --- | --- | --- | --- |
|  | Mean | Std. Deviation | N |
| HflexBase_time | 15.13 | 15.995 | 15 |
| HflexPlus_time | 15.40 | 16.322 | 15 |
| HflexMinus_time | 14.00 | 16.920 | 15 |

| **Tests of Within-Subjects Effects** | | | | | | | |
| --- | --- | --- | --- | --- | --- | --- | --- |
| Measure: MEASURE_1 | | | | | | | |
| Source | | Type III Sum of Squares | df | Mean Square | F | Sig. | Partial Eta Squared |
| FPA | Sphericity Assumed | 16.578 | 2 | 8.289 | .505 | .609 | .035 |
|  | Greenhouse-Geisser | 16.578 | 1.031 | 16.073 | .505 | .494 | .035 |
|  | Huynh-Feldt | 16.578 | 1.039 | 15.960 | .505 | .495 | .035 |
|  | Lower-bound | 16.578 | 1.000 | 16.578 | .505 | .489 | .035 |
| Error(FPA) | Sphericity Assumed | 459.422 | 28 | 16.408 |  |  |  |
|  | Greenhouse-Geisser | 459.422 | 14.439 | 31.817 |  |  |  |
|  | Huynh-Feldt | 459.422 | 14.542 | 31.592 |  |  |  |
|  | Lower-bound | 459.422 | 14.000 | 32.816 |  |  |  |

| **Pairwise Comparisons** | | | | | | |
| --- | --- | --- | --- | --- | --- | --- |
| Measure: MEASURE_1 | | | | | | |
| (I) FPA | (J) FPA | Mean Difference (I-J) | Std. Error | Sig.^a^ | 95% Confidence Interval for Difference^a^ | |
|  |  |  |  |  | Lower Bound | Upper Bound |
| 1 | 2 | -.267 | .300 | 1.000 | -1.083 | .549 |
|  | 3 | 1.133 | 1.872 | 1.000 | -3.954 | 6.220 |
| 2 | 1 | .267 | .300 | 1.000 | -.549 | 1.083 |
|  | 3 | 1.400 | 1.723 | 1.000 | -3.283 | 6.083 |
| 3 | 1 | -1.133 | 1.872 | 1.000 | -6.220 | 3.954 |
|  | 2 | -1.400 | 1.723 | 1.000 | -6.083 | 3.283 |
| Based on estimated marginal means | | | | | | |
| a. Adjustment for multiple comparisons: Bonferroni. | | | | | | |

**C. Hip flexion at touchdown**

| **Within-Subjects Factors** | |
| --- | --- |
| Measure: MEASURE_1 | |
| FPA | Dependent Variable |
| 1 | HflexBase_TD |
| 2 | HflexPlus_TD |
| 3 | HflexMinus_TD |

| **Descriptive Statistics** | | | |
| --- | --- | --- | --- |
|  | Mean | Std. Deviation | N |
| HflexBase_TD | 35.3298 | 4.95498 | 15 |
| HflexPlus_TD | 35.7694 | 5.07157 | 15 |
| HflexMinus_TD | 35.3806 | 4.75124 | 15 |

| **Tests of Within-Subjects Effects** | | | | | | | |
| --- | --- | --- | --- | --- | --- | --- | --- |
| Measure: MEASURE_1 | | | | | | | |
| Source | | Type III Sum of Squares | df | Mean Square | F | Sig. | Partial Eta Squared |
| FPA | Sphericity Assumed | 1.735 | 2 | .868 | .619 | .546 | .042 |
|  | Greenhouse-Geisser | 1.735 | 1.961 | .885 | .619 | .543 | .042 |
|  | Huynh-Feldt | 1.735 | 2.000 | .868 | .619 | .546 | .042 |
|  | Lower-bound | 1.735 | 1.000 | 1.735 | .619 | .445 | .042 |
| Error(FPA) | Sphericity Assumed | 39.270 | 28 | 1.402 |  |  |  |
|  | Greenhouse-Geisser | 39.270 | 27.460 | 1.430 |  |  |  |
|  | Huynh-Feldt | 39.270 | 28.000 | 1.402 |  |  |  |
|  | Lower-bound | 39.270 | 14.000 | 2.805 |  |  |  |

| **Pairwise Comparisons** | | | | | | |
| --- | --- | --- | --- | --- | --- | --- |
| Measure: MEASURE_1 | | | | | | |
| (I) FPA | (J) FPA | Mean Difference (I-J) | Std. Error | Sig.^a^ | 95% Confidence Interval for Difference^a^ | |
|  |  |  |  |  | Lower Bound | Upper Bound |
| 1 | 2 | -.440 | .401 | .875 | -1.530 | .651 |
|  | 3 | -.051 | .451 | 1.000 | -1.276 | 1.174 |
| 2 | 1 | .440 | .401 | .875 | -.651 | 1.530 |
|  | 3 | .389 | .444 | 1.000 | -.817 | 1.595 |
| 3 | 1 | .051 | .451 | 1.000 | -1.174 | 1.276 |
|  | 2 | -.389 | .444 | 1.000 | -1.595 | .817 |
| Based on estimated marginal means | | | | | | |
| a. Adjustment for multiple comparisons: Bonferroni. | | | | | | |

**D. Hip flexion excursion**

| **Within-Subjects Factors** | |
| --- | --- |
| Measure: MEASURE_1 | |
| FPA | Dependent Variable |
| 1 | HflexBase_excur |
| 2 | HflexPlus_excur |
| 3 | HflexMinus_excur |

| **Descriptive Statistics** | | | |
| --- | --- | --- | --- |
|  | Mean | Std. Deviation | N |
| HflexBase_excur | 1.4555 | 2.15529 | 15 |
| HflexPlus_excur | 1.2679 | 1.90128 | 15 |
| HflexMinus_excur | 1.1094 | 1.59530 | 15 |

| **Tests of Within-Subjects Effects** | | | | | | | |
| --- | --- | --- | --- | --- | --- | --- | --- |
| Measure: MEASURE_1 | | | | | | | |
| Source | | Type III Sum of Squares | df | Mean Square | F | Sig. | Partial Eta Squared |
| FPA | Sphericity Assumed | .901 | 2 | .450 | 1.494 | .242 | .096 |
|  | Greenhouse-Geisser | .901 | 1.453 | .620 | 1.494 | .245 | .096 |
|  | Huynh-Feldt | .901 | 1.578 | .571 | 1.494 | .245 | .096 |
|  | Lower-bound | .901 | 1.000 | .901 | 1.494 | .242 | .096 |
| Error(FPA) | Sphericity Assumed | 8.442 | 28 | .302 |  |  |  |
|  | Greenhouse-Geisser | 8.442 | 20.341 | .415 |  |  |  |
|  | Huynh-Feldt | 8.442 | 22.086 | .382 |  |  |  |
|  | Lower-bound | 8.442 | 14.000 | .603 |  |  |  |

| **Pairwise Comparisons** | | | | | | |
| --- | --- | --- | --- | --- | --- | --- |
| Measure: MEASURE_1 | | | | | | |
| (I) FPA | (J) FPA | Mean Difference (I-J) | Std. Error | Sig.^a^ | 95% Confidence Interval for Difference^a^ | |
|  |  |  |  |  | Lower Bound | Upper Bound |
| 1 | 2 | .188 | .127 | .489 | -.159 | .534 |
|  | 3 | .346 | .239 | .511 | -.304 | .997 |
| 2 | 1 | -.188 | .127 | .489 | -.534 | .159 |
|  | 3 | .158 | .217 | 1.000 | -.431 | .748 |
| 3 | 1 | -.346 | .239 | .511 | -.997 | .304 |
|  | 2 | -.158 | .217 | 1.000 | -.748 | .431 |
| Based on estimated marginal means | | | | | | |
| a. Adjustment for multiple comparisons: Bonferroni. | | | | | | |
