# Supplementary material for: The effect of changing foot progression angle using real-time visual feedback on rearfoot eversion during running
Source: PLoS One. 2021 Feb 10;16(2):e0246425. doi: 10.1371/journal.pone.0246425 (PMC7875396; doi:10.1371/journal.pone.0246425)
Supplement: S8 Fig — (DOCX) [file pone.0246425.s008.docx]

**S8 Fig**. One-way repeated measure ANOVA results for knee flexion

**A. Peak knee flexion**

| **Within-Subjects Factors** | |
| --- | --- |
| Measure: MEASURE_1 | |
| FPA | Dependent Variable |
| 1 | KflexBase_peak |
| 2 | KflexPlus_peak |
| 3 | KflexMinus_peak |

| **Descriptive Statistics** | | | |
| --- | --- | --- | --- |
|  | Mean | Std. Deviation | N |
| KflexBase_peak | 41.4836 | 4.45629 | 15 |
| KflexPlus_peak | 40.0076 | 4.57107 | 15 |
| KflexMinus_peak | 40.1811 | 4.85809 | 15 |

| **Tests of Within-Subjects Effects** | | | | | | | |
| --- | --- | --- | --- | --- | --- | --- | --- |
| Measure: MEASURE_1 | | | | | | | |
| Source | | Type III Sum of Squares | df | Mean Square | F | Sig. | Partial Eta Squared |
| FPA | Sphericity Assumed | 19.526 | 2 | 9.763 | 8.810 | .001 | .386 |
|  | Greenhouse-Geisser | 19.526 | 1.954 | 9.995 | 8.810 | .001 | .386 |
|  | Huynh-Feldt | 19.526 | 2.000 | 9.763 | 8.810 | .001 | .386 |
|  | Lower-bound | 19.526 | 1.000 | 19.526 | 8.810 | .010 | .386 |
| Error(FPA) | Sphericity Assumed | 31.028 | 28 | 1.108 |  |  |  |
|  | Greenhouse-Geisser | 31.028 | 27.350 | 1.134 |  |  |  |
|  | Huynh-Feldt | 31.028 | 28.000 | 1.108 |  |  |  |
|  | Lower-bound | 31.028 | 14.000 | 2.216 |  |  |  |

| **Pairwise Comparisons** | | | | | | |
| --- | --- | --- | --- | --- | --- | --- |
| Measure: MEASURE_1 | | | | | | |
| (I) FPA | (J) FPA | Mean Difference (I-J) | Std. Error | Sig.^b^ | 95% Confidence Interval for Difference^b^ | |
|  |  |  |  |  | Lower Bound | Upper Bound |
| 1 | 2 | 1.476^*^ | .356 | .003 | .509 | 2.443 |
|  | 3 | 1.303^*^ | .388 | .014 | .247 | 2.358 |
| 2 | 1 | -1.476^*^ | .356 | .003 | -2.443 | -.509 |
|  | 3 | -.173 | .407 | 1.000 | -1.280 | .933 |
| 3 | 1 | -1.303^*^ | .388 | .014 | -2.358 | -.247 |
|  | 2 | .173 | .407 | 1.000 | -.933 | 1.280 |
| Based on estimated marginal means | | | | | | |
| *. The mean difference is significant at the .05 level. | | | | | | |
| b. Adjustment for multiple comparisons: Bonferroni. | | | | | | |

**B. Time to peak knee flexion**

| **Within-Subjects Factors** | |
| --- | --- |
| Measure: MEASURE_1 | |
| FPA | Dependent Variable |
| 1 | KflexBase_time |
| 2 | KflexPlus_time |
| 3 | KflexMinus_time |

| **Descriptive Statistics** | | | |
| --- | --- | --- | --- |
|  | Mean | Std. Deviation | N |
| KflexBase_time | 48.07 | 1.944 | 15 |
| KflexPlus_time | 48.07 | 2.549 | 15 |
| KflexMinus_time | 48.60 | 1.404 | 15 |

| **Tests of Within-Subjects Effects** | | | | | | | |
| --- | --- | --- | --- | --- | --- | --- | --- |
| Measure: MEASURE_1 | | | | | | | |
| Source | | Type III Sum of Squares | df | Mean Square | F | Sig. | Partial Eta Squared |
| FPA | Sphericity Assumed | 2.844 | 2 | 1.422 | .833 | .445 | .056 |
|  | Greenhouse-Geisser | 2.844 | 1.848 | 1.539 | .833 | .438 | .056 |
|  | Huynh-Feldt | 2.844 | 2.000 | 1.422 | .833 | .445 | .056 |
|  | Lower-bound | 2.844 | 1.000 | 2.844 | .833 | .377 | .056 |
| Error(FPA) | Sphericity Assumed | 47.822 | 28 | 1.708 |  |  |  |
|  | Greenhouse-Geisser | 47.822 | 25.876 | 1.848 |  |  |  |
|  | Huynh-Feldt | 47.822 | 28.000 | 1.708 |  |  |  |
|  | Lower-bound | 47.822 | 14.000 | 3.416 |  |  |  |

| **Pairwise Comparisons** | | | | | | |
| --- | --- | --- | --- | --- | --- | --- |
| Measure: MEASURE_1 | | | | | | |
| (I) FPA | (J) FPA | Mean Difference (I-J) | Std. Error | Sig.^a^ | 95% Confidence Interval for Difference^a^ | |
|  |  |  |  |  | Lower Bound | Upper Bound |
| 1 | 2 | .000 | .414 | 1.000 | -1.125 | 1.125 |
|  | 3 | -.533 | .477 | .846 | -1.829 | .762 |
| 2 | 1 | .000 | .414 | 1.000 | -1.125 | 1.125 |
|  | 3 | -.533 | .533 | 1.000 | -1.983 | .916 |
| 3 | 1 | .533 | .477 | .846 | -.762 | 1.829 |
|  | 2 | .533 | .533 | 1.000 | -.916 | 1.983 |
| Based on estimated marginal means | | | | | | |
| a. Adjustment for multiple comparisons: Bonferroni. | | | | | | |

**C. Knee flexion at touchdown**

| **Within-Subjects Factors** | |
| --- | --- |
| Measure: MEASURE_1 | |
| FPA | Dependent Variable |
| 1 | KflexBase_TD |
| 2 | KflexPlus_TD |
| 3 | KflexMinus_TD |

| **Descriptive Statistics** | | | |
| --- | --- | --- | --- |
|  | Mean | Std. Deviation | N |
| KflexBase_TD | 9.3359 | 4.23665 | 15 |
| KflexPlus_TD | 9.8516 | 4.21342 | 15 |
| KflexMinus_TD | 10.4199 | 3.76552 | 15 |

| **Tests of Within-Subjects Effects** | | | | | | | |
| --- | --- | --- | --- | --- | --- | --- | --- |
| Measure: MEASURE_1 | | | | | | | |
| Source | | Type III Sum of Squares | df | Mean Square | F | Sig. | Partial Eta Squared |
| FPA | Sphericity Assumed | 8.820 | 2 | 4.410 | 1.829 | .179 | .116 |
|  | Greenhouse-Geisser | 8.820 | 1.579 | 5.584 | 1.829 | .188 | .116 |
|  | Huynh-Feldt | 8.820 | 1.746 | 5.051 | 1.829 | .185 | .116 |
|  | Lower-bound | 8.820 | 1.000 | 8.820 | 1.829 | .198 | .116 |
| Error(FPA) | Sphericity Assumed | 67.501 | 28 | 2.411 |  |  |  |
|  | Greenhouse-Geisser | 67.501 | 22.112 | 3.053 |  |  |  |
|  | Huynh-Feldt | 67.501 | 24.449 | 2.761 |  |  |  |
|  | Lower-bound | 67.501 | 14.000 | 4.821 |  |  |  |

| **Pairwise Comparisons** | | | | | | |
| --- | --- | --- | --- | --- | --- | --- |
| Measure: MEASURE_1 | | | | | | |
| (I) FPA | (J) FPA | Mean Difference (I-J) | Std. Error | Sig.^a^ | 95% Confidence Interval for Difference^a^ | |
|  |  |  |  |  | Lower Bound | Upper Bound |
| 1 | 2 | -.516 | .459 | .841 | -1.764 | .733 |
|  | 3 | -1.084 | .696 | .424 | -2.974 | .806 |
| 2 | 1 | .516 | .459 | .841 | -.733 | 1.764 |
|  | 3 | -.568 | .519 | .876 | -1.979 | .842 |
| 3 | 1 | 1.084 | .696 | .424 | -.806 | 2.974 |
|  | 2 | .568 | .519 | .876 | -.842 | 1.979 |
| Based on estimated marginal means | | | | | | |
| a. Adjustment for multiple comparisons: Bonferroni. | | | | | | |

**D. Knee flexion excursion**

| **Within-Subjects Factors** | |
| --- | --- |
| Measure: MEASURE_1 | |
| FPA | Dependent Variable |
| 1 | KflexBase_excur |
| 2 | KflexPlus_excur |
| 3 | KflexMinus_excur |

| **Descriptive Statistics** | | | |
| --- | --- | --- | --- |
|  | Mean | Std. Deviation | N |
| KflexBase_excur | 32.1477 | 5.47913 | 15 |
| KflexPlus_excur | 30.1560 | 4.29058 | 15 |
| KflexMinus_excur | 29.7612 | 4.70751 | 15 |

| **Tests of Within-Subjects Effects** | | | | | | | |
| --- | --- | --- | --- | --- | --- | --- | --- |
| Measure: MEASURE_1 | | | | | | | |
| Source | | Type III Sum of Squares | df | Mean Square | F | Sig. | Partial Eta Squared |
| FPA | Sphericity Assumed | 49.092 | 2 | 24.546 | 11.461 | .000 | .450 |
|  | Greenhouse-Geisser | 49.092 | 1.772 | 27.697 | 11.461 | .000 | .450 |
|  | Huynh-Feldt | 49.092 | 2.000 | 24.546 | 11.461 | .000 | .450 |
|  | Lower-bound | 49.092 | 1.000 | 49.092 | 11.461 | .004 | .450 |
| Error(FPA) | Sphericity Assumed | 59.966 | 28 | 2.142 |  |  |  |
|  | Greenhouse-Geisser | 59.966 | 24.814 | 2.417 |  |  |  |
|  | Huynh-Feldt | 59.966 | 28.000 | 2.142 |  |  |  |
|  | Lower-bound | 59.966 | 14.000 | 4.283 |  |  |  |

| **Pairwise Comparisons** | | | | | | |
| --- | --- | --- | --- | --- | --- | --- |
| Measure: MEASURE_1 | | | | | | |
| (I) FPA | (J) FPA | Mean Difference (I-J) | Std. Error | Sig.^b^ | 95% Confidence Interval for Difference^b^ | |
|  |  |  |  |  | Lower Bound | Upper Bound |
| 1 | 2 | 1.992^*^ | .471 | .003 | .711 | 3.273 |
|  | 3 | 2.387^*^ | .622 | .005 | .696 | 4.077 |
| 2 | 1 | -1.992^*^ | .471 | .003 | -3.273 | -.711 |
|  | 3 | .395 | .498 | 1.000 | -.958 | 1.747 |
| 3 | 1 | -2.387^*^ | .622 | .005 | -4.077 | -.696 |
|  | 2 | -.395 | .498 | 1.000 | -1.747 | .958 |
| Based on estimated marginal means | | | | | | |
| *. The mean difference is significant at the .05 level. | | | | | | |
| b. Adjustment for multiple comparisons: Bonferroni. | | | | | | |
