# Supplementary material for: The effect of changing foot progression angle using real-time visual feedback on rearfoot eversion during running
Source: PLoS One. 2021 Feb 10;16(2):e0246425. doi: 10.1371/journal.pone.0246425 (PMC7875396; doi:10.1371/journal.pone.0246425)
Supplement: S9 Fig — (DOCX) [file pone.0246425.s009.docx]

**S9 Fig**. One-way repeated measure ANOVA results for Ankle dorsiflexion

**A. Peak ankle dorsiflexion**

| **Within-Subjects Factors** | |
| --- | --- |
| Measure: MEASURE_1 | |
| FPA | Dependent Variable |
| 1 | AflexBase_peak |
| 2 | AflexPlus_peak |
| 3 | AflexMinus_peak |

| **Descriptive Statistics** | | | |
| --- | --- | --- | --- |
|  | Mean | Std. Deviation | N |
| AflexBase_peak | 23.9852 | 3.74807 | 15 |
| AflexPlus_peak | 22.3722 | 3.58385 | 15 |
| AflexMinus_peak | 23.4041 | 3.69499 | 15 |

| **Tests of Within-Subjects Effects** | | | | | | | |
| --- | --- | --- | --- | --- | --- | --- | --- |
| Measure: MEASURE_1 | | | | | | | |
| Source | | Type III Sum of Squares | df | Mean Square | F | Sig. | Partial Eta Squared |
| FPA | Sphericity Assumed | 20.021 | 2 | 10.010 | 10.278 | .000 | .423 |
|  | Greenhouse-Geisser | 20.021 | 1.519 | 13.177 | 10.278 | .002 | .423 |
|  | Huynh-Feldt | 20.021 | 1.666 | 12.018 | 10.278 | .001 | .423 |
|  | Lower-bound | 20.021 | 1.000 | 20.021 | 10.278 | .006 | .423 |
| Error(FPA) | Sphericity Assumed | 27.270 | 28 | .974 |  |  |  |
|  | Greenhouse-Geisser | 27.270 | 21.272 | 1.282 |  |  |  |
|  | Huynh-Feldt | 27.270 | 23.322 | 1.169 |  |  |  |
|  | Lower-bound | 27.270 | 14.000 | 1.948 |  |  |  |

| **Pairwise Comparisons** | | | | | | |
| --- | --- | --- | --- | --- | --- | --- |
| Measure: MEASURE_1 | | | | | | |
| (I) FPA | (J) FPA | Mean Difference (I-J) | Std. Error | Sig.^b^ | 95% Confidence Interval for Difference^b^ | |
|  |  |  |  |  | Lower Bound | Upper Bound |
| 1 | 2 | 1.613^*^ | .314 | .000 | .759 | 2.467 |
|  | 3 | .581 | .450 | .653 | -.642 | 1.805 |
| 2 | 1 | -1.613^*^ | .314 | .000 | -2.467 | -.759 |
|  | 3 | -1.032^*^ | .297 | .011 | -1.839 | -.225 |
| 3 | 1 | -.581 | .450 | .653 | -1.805 | .642 |
|  | 2 | 1.032^*^ | .297 | .011 | .225 | 1.839 |
| Based on estimated marginal means | | | | | | |
| *. The mean difference is significant at the .05 level. | | | | | | |
| b. Adjustment for multiple comparisons: Bonferroni. | | | | | | |

**B. Time to peak ankle dorsiflexion**

| **Within-Subjects Factors** | |
| --- | --- |
| Measure: MEASURE_1 | |
| FPA | Dependent Variable |
| 1 | AflexBase_peak |
| 2 | AflexPlus_peak |
| 3 | AflexMinus_peak |

| **Descriptive Statistics** | | | |
| --- | --- | --- | --- |
|  | Mean | Std. Deviation | N |
| AflexBase_peak | 23.9852 | 3.74807 | 15 |
| AflexPlus_peak | 22.3722 | 3.58385 | 15 |
| AflexMinus_peak | 23.4041 | 3.69499 | 15 |

| **Tests of Within-Subjects Effects** | | | | | | | |
| --- | --- | --- | --- | --- | --- | --- | --- |
| Measure: MEASURE_1 | | | | | | | |
| Source | | Type III Sum of Squares | df | Mean Square | F | Sig. | Partial Eta Squared |
| FPA | Sphericity Assumed | 20.021 | 2 | 10.010 | 10.278 | .000 | .423 |
|  | Greenhouse-Geisser | 20.021 | 1.519 | 13.177 | 10.278 | .002 | .423 |
|  | Huynh-Feldt | 20.021 | 1.666 | 12.018 | 10.278 | .001 | .423 |
|  | Lower-bound | 20.021 | 1.000 | 20.021 | 10.278 | .006 | .423 |
| Error(FPA) | Sphericity Assumed | 27.270 | 28 | .974 |  |  |  |
|  | Greenhouse-Geisser | 27.270 | 21.272 | 1.282 |  |  |  |
|  | Huynh-Feldt | 27.270 | 23.322 | 1.169 |  |  |  |
|  | Lower-bound | 27.270 | 14.000 | 1.948 |  |  |  |

| **Pairwise Comparisons** | | | | | | |
| --- | --- | --- | --- | --- | --- | --- |
| Measure: MEASURE_1 | | | | | | |
| (I) FPA | (J) FPA | Mean Difference (I-J) | Std. Error | Sig.^b^ | 95% Confidence Interval for Difference^b^ | |
|  |  |  |  |  | Lower Bound | Upper Bound |
| 1 | 2 | 1.613^*^ | .314 | .000 | .759 | 2.467 |
|  | 3 | .581 | .450 | .653 | -.642 | 1.805 |
| 2 | 1 | -1.613^*^ | .314 | .000 | -2.467 | -.759 |
|  | 3 | -1.032^*^ | .297 | .011 | -1.839 | -.225 |
| 3 | 1 | -.581 | .450 | .653 | -1.805 | .642 |
|  | 2 | 1.032^*^ | .297 | .011 | .225 | 1.839 |
| Based on estimated marginal means | | | | | | |
| *. The mean difference is significant at the .05 level. | | | | | | |
| b. Adjustment for multiple comparisons: Bonferroni. | | | | | | |

**C. Ankle dorsiflexion at touchdown**

| **Within-Subjects Factors** | |
| --- | --- |
| Measure: MEASURE_1 | |
| FPA | Dependent Variable |
| 1 | AflexBase_TD |
| 2 | AflexPlus_TD |
| 3 | AflexMinus_TD |

| **Descriptive Statistics** | | | |
| --- | --- | --- | --- |
|  | Mean | Std. Deviation | N |
| AflexBase_TD | 5.2596 | 3.89237 | 15 |
| AflexPlus_TD | 5.4143 | 2.86485 | 15 |
| AflexMinus_TD | 5.2986 | 3.69906 | 15 |

| **Tests of Within-Subjects Effects** | | | | | | | |
| --- | --- | --- | --- | --- | --- | --- | --- |
| Measure: MEASURE_1 | | | | | | | |
| Source | | Type III Sum of Squares | df | Mean Square | F | Sig. | Partial Eta Squared |
| FPA | Sphericity Assumed | .194 | 2 | .097 | .077 | .927 | .005 |
|  | Greenhouse-Geisser | .194 | 1.767 | .110 | .077 | .907 | .005 |
|  | Huynh-Feldt | .194 | 2.000 | .097 | .077 | .927 | .005 |
|  | Lower-bound | .194 | 1.000 | .194 | .077 | .786 | .005 |
| Error(FPA) | Sphericity Assumed | 35.517 | 28 | 1.268 |  |  |  |
|  | Greenhouse-Geisser | 35.517 | 24.743 | 1.435 |  |  |  |
|  | Huynh-Feldt | 35.517 | 28.000 | 1.268 |  |  |  |
|  | Lower-bound | 35.517 | 14.000 | 2.537 |  |  |  |

| **Pairwise Comparisons** | | | | | | |
| --- | --- | --- | --- | --- | --- | --- |
| Measure: MEASURE_1 | | | | | | |
| (I) FPA | (J) FPA | Mean Difference (I-J) | Std. Error | Sig.^a^ | 95% Confidence Interval for Difference^a^ | |
|  |  |  |  |  | Lower Bound | Upper Bound |
| 1 | 2 | -.155 | .344 | 1.000 | -1.090 | .781 |
|  | 3 | -.039 | .405 | 1.000 | -1.141 | 1.063 |
| 2 | 1 | .155 | .344 | 1.000 | -.781 | 1.090 |
|  | 3 | .116 | .474 | 1.000 | -1.172 | 1.403 |
| 3 | 1 | .039 | .405 | 1.000 | -1.063 | 1.141 |
|  | 2 | -.116 | .474 | 1.000 | -1.403 | 1.172 |
| Based on estimated marginal means | | | | | | |
| a. Adjustment for multiple comparisons: Bonferroni. | | | | | | |

**D. Ankle dorsiflexion excursion**

| **Within-Subjects Factors** | |
| --- | --- |
| Measure: MEASURE_1 | |
| FPA | Dependent Variable |
| 1 | AflexBase_excur |
| 2 | AflexPlus_excur |
| 3 | AflexMinus_excur |

| **Descriptive Statistics** | | | |
| --- | --- | --- | --- |
|  | Mean | Std. Deviation | N |
| AflexBase_excur | 18.7257 | 2.52894 | 15 |
| AflexPlus_excur | 16.9580 | 2.70880 | 15 |
| AflexMinus_excur | 18.1055 | 3.12827 | 15 |

| **Tests of Within-Subjects Effects** | | | | | | | |
| --- | --- | --- | --- | --- | --- | --- | --- |
| Measure: MEASURE_1 | | | | | | | |
| Source | | Type III Sum of Squares | df | Mean Square | F | Sig. | Partial Eta Squared |
| FPA | Sphericity Assumed | 24.131 | 2 | 12.065 | 5.500 | .010 | .282 |
|  | Greenhouse-Geisser | 24.131 | 1.615 | 14.944 | 5.500 | .016 | .282 |
|  | Huynh-Feldt | 24.131 | 1.794 | 13.450 | 5.500 | .013 | .282 |
|  | Lower-bound | 24.131 | 1.000 | 24.131 | 5.500 | .034 | .282 |
| Error(FPA) | Sphericity Assumed | 61.418 | 28 | 2.194 |  |  |  |
|  | Greenhouse-Geisser | 61.418 | 22.606 | 2.717 |  |  |  |
|  | Huynh-Feldt | 61.418 | 25.118 | 2.445 |  |  |  |
|  | Lower-bound | 61.418 | 14.000 | 4.387 |  |  |  |

| **Pairwise Comparisons** | | | | | | |
| --- | --- | --- | --- | --- | --- | --- |
| Measure: MEASURE_1 | | | | | | |
| (I) FPA | (J) FPA | Mean Difference (I-J) | Std. Error | Sig.^b^ | 95% Confidence Interval for Difference^b^ | |
|  |  |  |  |  | Lower Bound | Upper Bound |
| 1 | 2 | 1.768^*^ | .390 | .001 | .708 | 2.828 |
|  | 3 | .620 | .583 | .916 | -.963 | 2.204 |
| 2 | 1 | -1.768^*^ | .390 | .001 | -2.828 | -.708 |
|  | 3 | -1.148 | .621 | .258 | -2.836 | .541 |
| 3 | 1 | -.620 | .583 | .916 | -2.204 | .963 |
|  | 2 | 1.148 | .621 | .258 | -.541 | 2.836 |
| Based on estimated marginal means | | | | | | |
| *. The mean difference is significant at the .05 level. | | | | | | |
| b. Adjustment for multiple comparisons: Bonferroni. | | | | | | |
