# Supplementary material for: The effect of changing foot progression angle using real-time visual feedback on rearfoot eversion during running
Source: PLoS One. 2021 Feb 10;16(2):e0246425. doi: 10.1371/journal.pone.0246425 (PMC7875396; doi:10.1371/journal.pone.0246425)
Supplement: S10 Fig — (DOCX) [file pone.0246425.s010.docx]

**S10 Fig**. One-way repeated measure ANOVA results for peak ankle power

| **Within-Subjects Factors** | |
| --- | --- |
| Measure: MEASURE_1 | |
| FPA | Dependent Variable |
| 1 | ApowerBase_peak |
| 2 | ApowerPlus_peak |
| 3 | ApowerMinus_peak |

| **Descriptive Statistics** | | | |
| --- | --- | --- | --- |
|  | Mean | Std. Deviation | N |
| ApowerBase_peak | 13.6881 | 2.45800 | 15 |
| ApowerPlus_peak | 12.3482 | 1.98814 | 15 |
| ApowerMinus_peak | 13.2609 | 2.45385 | 15 |

| **Tests of Within-Subjects Effects** | | | | | | | |
| --- | --- | --- | --- | --- | --- | --- | --- |
| Measure: MEASURE_1 | | | | | | | |
| Source | | Type III Sum of Squares | df | Mean Square | F | Sig. | Partial Eta Squared |
| FPA | Sphericity Assumed | 14.054 | 2 | 7.027 | 11.726 | .000 | .456 |
|  | Greenhouse-Geisser | 14.054 | 1.818 | 7.731 | 11.726 | .000 | .456 |
|  | Huynh-Feldt | 14.054 | 2.000 | 7.027 | 11.726 | .000 | .456 |
|  | Lower-bound | 14.054 | 1.000 | 14.054 | 11.726 | .004 | .456 |
| Error(FPA) | Sphericity Assumed | 16.779 | 28 | .599 |  |  |  |
|  | Greenhouse-Geisser | 16.779 | 25.451 | .659 |  |  |  |
|  | Huynh-Feldt | 16.779 | 28.000 | .599 |  |  |  |
|  | Lower-bound | 16.779 | 14.000 | 1.199 |  |  |  |

| **Pairwise Comparisons** | | | | | | |
| --- | --- | --- | --- | --- | --- | --- |
| Measure: MEASURE_1 | | | | | | |
| (I) FPA | (J) FPA | Mean Difference (I-J) | Std. Error | Sig.^b^ | 95% Confidence Interval for Difference^b^ | |
|  |  |  |  |  | Lower Bound | Upper Bound |
| 1 | 2 | 1.340^*^ | .290 | .001 | .551 | 2.129 |
|  | 3 | .427 | .315 | .590 | -.429 | 1.283 |
| 2 | 1 | -1.340^*^ | .290 | .001 | -2.129 | -.551 |
|  | 3 | -.913^*^ | .237 | .005 | -1.557 | -.269 |
| 3 | 1 | -.427 | .315 | .590 | -1.283 | .429 |
|  | 2 | .913^*^ | .237 | .005 | .269 | 1.557 |
| Based on estimated marginal means | | | | | | |
| *. The mean difference is significant at the .05 level. | | | | | | |
| b. Adjustment for multiple comparisons: Bonferroni. | | | | | | |
